# Microarray patch vaccines for typhoid conjugate vaccines: A global cost-effectiveness analysis

**DOI:** 10.1016/j.vaccine.2025.127055

**Published:** 2025-04-19

**Authors:** Marina Antillon, Anna Verjans, Fayad El Sheikh, Tiziana Scarna, Mercy Mvundura

**Affiliations:** a Swiss Tropical and Public Health Institute, Kreuzstrasse 2, 4123 Allschwil, Switzerland; b University of Basel, Petersplatz 1, P. O. Box, 4001, Basel, Switzerland; c Gavi, The Vaccine Alliance, Global Health Campus, Chemin du Pommier 40, 1218 Le Grand-Saconnex, Geneva, Switzerland; d PATH, 2201 Westlake Ave, Suite 200, Seattle, United States

**Keywords:** Typhoid conjugate vaccine, Cost-effectiveness, Modeling, Economic evaluation, Microarray patches, Value proposition.

## Abstract

A novel typhoid conjugate vaccine (TCV) presentation, the microarray patch (MAP), is in early-stage development and could potentially help to increase coverage in hard-to-reach populations beyond what is being achieved with the current TCV in a vial presentation administered with a needle and syringe (TCV-N&S). However, TCV-MAPs may come at a higher price per dose than TCV-N&S. Our analysis evaluated the potential cost-effectiveness of TCV-MAPs alongside TCV-N&S compared to TCV-N&S alone.

A global extended cost-effectiveness analysis, taking a health care perspective, was conducted for 133 low- to upper-middle-income countries for a time horizon of 20 years (2033–2052). Health outcomes were expressed in disability-adjusted life years (DALYs) and costs in 2021 US dollars, both discounted at 3 %. We assumed TCV-MAP would cost 1.33 to 3 times the price of the TCV-N&S vaccine. We calculated incremental cost-effectiveness ratios and evaluated them against various cost-effectiveness thresholds. For five selected countries, we conducted an additional subnational analysis to understand the potential value of a district-specific TCV-MAP implementation instead of a national rollout.

Across the 133 low- to upper-middle-income countries, national rollout of TCV-MAPs could avert an additional 5.2 million cases, 47,000 deaths, and 2.4 million DALYs compared to TCV-N&S only, at an additional cost of US$3.5 billion over 20 years. The largest proportion of the averted burden would be in the sub-Saharan African region. TCV-MAPs could be cost-effective in 33 % of the countries but in 78 % of sub-Saharan African countries. A subnational implementation could benefit some countries for which a national implementation may not be cost-effective, averting 2–15 % of cases for less than 1–3 % of the additional cost as compared to a national rollout. MAP price was a key driver of the results.

Regional or subnational implementation, coupled with a lower price point, could significantly improve the TCV-MAP value proposition.

## 1 Introduction

Typhoid fever, a bacterial disease caused by *Salmonella* serotype Typhi, affects 9 million people globally and causes over 100,000 deaths every year [1]. Unsafe drinking water and poor sanitary conditions are the key risk factors, making it a significant public health concern in low- and middle-income countries (LMICs), which bear 99 % of the burden [2]. The unequal distribution of these risk factors suggests potential disparities in the disease burden across socioeconomic strata [3,4]. Additionally, the global burden of typhoid is expected to increase due to climate change and urbanization [1]. Beyond its direct health impact on individuals, typhoid fever imposes significant economic costs and societal burdens, particularly in LMICs [[5], [6], [7], [8], [9], [10]]. In recent years, treatment has become more challenging due to the emergence of antimicrobial-resistant (AMR) strains, with multidrug-resistant (MDR) and extensively drug-resistant (XDR) cases on the rise in both endemic and non-endemic countries [11]. Given these challenges, vaccination remains a key strategy for reducing the spread of typhoid, with the potential to prevent cases and save lives.

While typhoid vaccines have existed for a long time, they have primarily been polysaccharide and live attenuated vaccines mainly targeted for travelers to endemic countries. The recent advent of novel typhoid conjugate vaccines (TCVs) has renewed hope in the fight against the pathogen, as these vaccines target infants and children in endemic countries. These vaccines offer improved immunogenicity in infants and longer-lasting protection compared to previous formulations [12]. As of May 2024, TCVs have been integrated into national expanded programs on immunization (EPI) in five Gavi-eligible countries (Pakistan, Liberia, Zimbabwe, Malawi, and Nepal) and two non-eligible countries (Samoa and Fiji) to be co-administered with the first dose of measles-containing vaccine (MCV) (usually at 9 months of age) or the second dose of measles-containing vaccine (usually at 15 months of age) [13,14]. However, despite the advances of novel TCVs, disparities in vaccine reach by wealth quintiles could hinder the capacity of TCVs to reduce disparities in disease burden across socioeconomic strata arising from unequal access to sanitation infrastructure [15].

Advances in vaccine delivery technology, such as the development of microarray patches (MAPs), which consist of microscopic projections that penetrate the outermost layer of the skin and are applied like a bandage, could pave the way for innovative approaches to vaccine administration in the next few years. MAPs have the potential to simplify vaccine delivery, reduce cold chain storage requirements due to improved thermostability, and enable vaccination by community health workers [16]. These characteristics could help extend the reach and coverage of vaccines beyond what is currently achieved by the available TCV presentation. Specifically, MAPs could be used for vaccination in hard-to-reach populations and could help reduce zero-dose and under-immunized children. While child vaccination could be the largest market share for TCV-MAPs, similar to TCV-N&S, MAPs are also envisioned to be used among travelers and military personnel, who would benefit from the greater convenience of a MAP. MAP presentations for several vaccines, including measles-rubella (MR), human papillomavirus (HPV), influenza, and rabies are currently at various stages of development [17], with the MR MAP being the most advanced in phase 2 clinical trials, while the TCV-MAP is in pre-clinical development [18,19].

This analysis presents the findings of an extended cost-effectiveness analysis (CEA) of a novel TCV presented in a new delivery technology of a MAP, compared to the currently available TCV in a vial presentation that is administered using a needle and syringe (TCV-N&S). The results from our analysis will provide insights to stakeholders on which use cases for MAPs could have the best value for money.

## 2 Materials and methods

This study assessed the impact, costs, and cost-effectiveness of the addition of TCV-MAPs following TCV-N&S introduction over a time horizon of 20 years (2033–2052) from the health care perspective. It is anticipated that TCV-MAPs will be available on the market by 2033. The analysis included 133 low-, lower-middle-, and upper-middle-income countries that have or could potentially introduce TCVs into their routine immunization programs. In five purposively selected countries where we had access to subnational data on key variables needed for the analysis, we assessed the potential cost-effectiveness of using TCV-MAPs at a subnational level.

### 2.1 Product profiles for TCV-MAPs and TCV-N&S

The TCV product profiles used in this analysis are based on the characteristics and attributes of various MAP vaccines currently in development, as well as the TCV-N&S vaccine available on the market. In this analysis, we assessed three potential TCV-MAP product profiles, each characterized by different attributes related to cold chain storage volume and the human resource time required for vaccine administration. The human resource time for vaccine administration encompasses both the time spent by the vaccinator administering the vaccine and the MAP wear time, which is the additional time spent observing the child after vaccination.

The specifications for the TCV product profiles used in this analysis are detailed below and also outlined in Table A-1 in Appendix A-1:

1. Baseline profile: has a volume of 20 cm^3^ and requires 70 s of human resource time for administration.
2. Pessimistic profile: has a volume of 20 cm^3^ and requires 5 min of human resource time for administration.
3. Optimistic profile: has a volume of 5 cm^3^ and requires 15 s of human resource time for administration.

Each of these three TCV-MAP profiles is compared to the TCV-N&S presentation, which is supplied in a 5-dose vial with a cold chain volume of 2.9 cm^3^ and an administration time of 17 s. For the TCV-N&S, there is no assumption that the infant must be observed after vaccination, thus, the listed time reflects only the vaccine administration time. The TCV N&S is WHO-prequalified for use in a controlled temperature chain (CTC), which allows storage of the vaccine at temperatures not exceeding 40 °C for up to 7 days or at temperatures not exceeding 55 °C for up to 3 days, just before administration. For TCV MAPs, we assumed stability profiles similar to TCV-N&S for the baseline and pessimistic MAP profiles. However, for the optimistic profile, we assumed enhanced thermostability with CTC use at temperatures below 40 °C for at least 2 months, based on the targeted profile.

The price per dose for TCV-N&S is $1.50, which is the 2018 to 2025 procurement price published by UNICEF [20]. An autodisable N&S, which is priced at $0.06, is also required for administration. [21]. Given that the price of TCV-MAPs is unknown, as the product is still under development, we evaluated four plausible price points: $2.00, $2.25, $3.00, and $4.50 per dose, representing price premiums of 1.33, 1.5, 2, and 3 times the vial presentation, respectively. The default or baseline price for a MAP was assumed at $3. We assumed non-inferiority of the MAP to the current TCV-N&S and no change in the duration of protection.

#### 2.1.1 Use cases

Based on the framework developed by Soble et al. [22], we assumed different use cases for TCV-MAPs according to three dimensions: (i) delivery setting, (ii) target population, and (iii) administrator:

1. Infants under two years of age vaccinated at fixed health posts, where routine immunization services are provided (UC1).
2. Infants vaccinated through outreach activities (UC2). Outreach activities refer to regular vaccination activities conducted within 5–15 km of a clinic, with health personnel from the facility traveling with a passive cold chain (vaccine carriers).
3. Infants vaccinated through mobile activities (UC3), targeting transient or hard-to-reach populations, such as nomadic communities or areas lacking permanent health infrastructure. Mobile activities refer to vaccination activities conducted more than 15 km from a clinic, with health personnel traveling with a more extensive passive cold chain.
4. Children aged 2 to 15 years vaccinated in a single TCV-MAP campaign at the introduction of TCV into the routine immunization program (UC4).
5. Military personnel over 15 years of age vaccinated at fixed military facilities (UC5).
6. Adult travelers over 15 years of age vaccinated at fixed travel clinics (UC6).

To evaluate each MAP profile relative to vaccination with the vial presentation, we considered three scenarios where TCV-MAPs are used alongside TCV-N&S in some scenarios:

1. Using TCV-MAPs in 80 % of all populations currently vaccinated, with TCV-N&S covering the remaining 20 %, similar to the assumption used in the MAP measles-rubella demand forecast [23]. We also assumed as the default assumption across all scenarios that MAPs would reach 20 % of the currently unvaccinated populations. Sensitivity analyses will look at other coverage levels of the currently unvaccinated population.
2. A targeted introduction of TCV-MAPs, maintaining the use of TCV-N&S in fixed health posts (UC1) while adopting TCV-MAPs in outreach and mobile settings (UC2 and UC3). The resulting increase in coverage is assumed to be distributed proportionately between outreach and mobile settings based on the historical population reach.
3. A complete transition to TCV-MAPs with universal adoption across all use cases and populations.

The main analysis used the baseline TCV-MAP profile and assumed an 80 % replacement of TCV-N&S with TCV MAPs as the default scenario (scenario 1).

### 2.2 Transmission and disease model

To simulate the impact of introducing the TCV-MAP, we combined a dynamic susceptible-infected-recovered (SIR) model with a probability tree of disease outcomes (See Fig. A-1, Fig. A-2 and Fig. A-3 in Appendix A-2). To model transmission, we expanded an existing age-stratified SIR model of typhoid transmission [[24], [25], [26]] by incorporating household wealth quintile stratification (see Fig. A 2 in Appendix A-2). To model health outcomes and costs, the output from the dynamic model was used as an input for a probability tree (see Fig. A 3 in Appendix A-2).

Unlike other iterations of the transmission model [[26], [27], [28]], this version does not include a water compartment. Water transmission is rarely distinguishable from person-to-person transmission unless there are data on seasonal trends of disease that correlate with weather patterns [26]. Moreover, the evaluated interventions do not specifically target water-mediated transmission. In the dynamic model, the flow of individuals between compartments is mathematically represented by a system of ordinary differential equations, as shown in Table A 2 in Appendix A-2.

Typhoid incidence per age group was retrieved from a previous burden model and burden estimates from the Institute for Health Metrics and Evaluation [2,29]. These burden estimates were then used to estimate the effective transmission rate, as described in a prior publication [25].

To address inequities in typhoid exposure and access to vaccination, our analysis integrates equity considerations and typhoid risk through two key indicators, obtained from the Demographic and Health Surveys (DHS) and the Multiple Indicator Cluster Surveys (MICS):

1. The likelihood of an individual having access to improved sanitation that is not shared with other households. Fig. A 4 in Appendix A-2 shows the disparities in sanitation access among wealth quintiles.
2. The likelihood of a child being vaccinated on time (at 9 months of age) or being reached through outreach, mobile activities, or one-time campaigns. To measure this, we used the prevalence of the first dose of measles-containing vaccine (MCV1) at one year of age as a proxy indicator. Disparities in vaccine coverage among wealth quintiles are shown in Fig. A 5 in Appendix A-2.

Incorporating wealth quintile strata enabled us to allocate transmission rates based on exposure to unimproved sanitation and vaccination coverage for each wealth quintile (see Fig. A 6 in Appendix A-2). The typhoid incidence from the expanded model compared to the original model is presented in Fig. A 7 and Fig. A 8 in Appendix A-2. The resulting distribution of cases among wealth quintiles is shown in Fig. A 9 in Appendix A-2.

Vaccination was simulated using a transmission model that reached endemic equilibrium, given the lack of data to project changes over time, and was applied to the age strata of interest. Protection from vaccines results from two mechanisms: direct (due to vaccination) and indirect (or ‘herd’ immunity due to reduced transmission in the community). Fig. A 10 in Appendix A-2 illustrates the projected impact of TCV-MAPs on the number of cases, considering the vaccine impact of TCV-N&S up to 2032, waning immunity of TCV-N&S, and the addition of TCV-MAPs to the program.

Disease parameters for the model are summarized in Table A 3 in Appendix A-3 and listed for each country in Supplemental File 1.

### 2.3 Key assumptions about TCV-N&S and TCV-MAPs

Key assumptions of the product and the population are listed in Table 1. We assumed no improvements in infrastructure, water, sanitation, and hygiene over the analysis period.

**Table 1:** Key input parameters for global and subnational analyses.

**Parameter**	**TCV-N&S**	**TCV-MAP**	**Rationale, sensitivity analysisvalues, andor datasourcesfor the parameters**
*Introduction date*	2023–2032	2033–2042	Rationale: Projected for each country based on historical vaccine introduction trends, forecasted Gavi eligibility, and burden of disease, assuming all countries introduce TCV-N&S regardless of cost-effectiveness. Source:[21]
*Introduction strategy*	Routine immunization (RI) at 9 months and a catch-up campaign for 9 months to 15 years of age (Table A 4 Appendix A-4)	Routine immunization (RI) at 9 months	Sensitivity analyses: 1) Use of TCV-N&S at 9 months in RI only (i.e., no campaign) TCV-MAPs are introduced later in RI only. 2) Use of the TCV vial at 9 months in RI with a catch-up campaign. TCV-MAPs are introduced later in RI only. 3) Option 1 above, but assuming that RI takes place at 15 months of age, the time of the MCV2 vaccine. 4) Option 2 above, but assuming that RI takes place at 15 months of age, the time of the MCV2 vaccine.
*Coverage in routine immunization*	Total Population (range): At 9 months: 26.3–99.7% At 15 months: 9–93% By wealth quintile (WQ) (range): At 9 months: 16.5–100% At 15 months: 6.1–94.5%	Total Population (range): At 9 months: 26.3–99.7% At 15 months: 9–93% By wealth quintile (WQ) (range): At 9 months: 16.5–100% At 15 months: 6.1–94.5%	Sources: DHS or MICS data on MCV1 coverage (at 9 months) for 133 countries and MCV2 coverage (at 15 months) for 20 countries. For countries without MCV2 data, we assumed that coverage was 20 percentage points lower for MCV2 than MCV1. The difference of 20 percentage points was the average difference in the 20 countries with available data (see Figure A 12 in Appendix A-8).
*Coverage in campaign*	10 percentage points lower than routine coverage.	10 percentage points lower than routine coverage.	Assumption. Source: [24]
*Product profile*	5-dose vial with a volume of 14.5 cm^3^ per vial or 2.9 cm^3^/dose. It takes 17 seconds to administer one dose of TCV.	Base profile: a single-dose package with a volume of 20 cm^3^/dose and a MAP administration time of 70 seconds per dose.	Sensitivity analyses: 1) Optimistic profile: Single-dose package with a volume of 20 cm^3^/dose and a MAP administration time of 5 minutes per dose. 2) Pessimistic profile: Single-dose package with a volume of 5 cm^3^/dose and a MAP administration time of 15 seconds per dose.
*Price*	$1.50	$3.00 (baseline)	TCV-N&S price for WHO-prequalified vaccines procured through UNICEF and introduced at national level so far is $1.50 (Bharat Biotech). TCV-MAP pricing informed by price benchmarking and cost of goods sold assessment. Sensitivity analyses: additional prices for MAPs explored were $2.00, $2.25, and $4.50. Source: [31]
***Market penetration (current population reached)***			
*If only TCV-N&S is available*	100%	0%	Scenario analyses: "Targeted” adoption: The vial is used for children in use case 1 (fixed health posts), so there is 100% market penetration by the vial and 0% by MAPs. MAPs are used for all children (both new and previously reached populations) in use cases 2–4. Complete replacement: MAPs replace the vial as the product used for all TCV vaccinations across all populations.
*If TCV-N&S and TCV-MAP are available*	20% across use cases	80% across use cases	
***Coverage of populations currently unreached***			
*Only TCV-N&S available*	0%	0%	New populations reached are spread across the use cases (fixed health post, mobile, and outreach activities) using MAPs in proportion to the coverage handled by each use case (see Appendix A-2 for TCV product distribution). Scenario analyses: 10% and 30% additional coverage by TCV-MAPs (see Appendix A-7).
*TCV-vial and TCV-MAP available*	0%	20%	
***Effective coverage by use case***			
*Use case 1: fixed post*	30%–98%	30%–98%	Sources: [22] (see Supplementary File 1)
*Use case 2: outreach*	1%–65%	1%–65%	Sources: [22] (see Supplementary File 1)
*Use case 3: mobile*	1%–20%	1%–20%	Sources: [22] (see Supplementary File 1)
*Use case 4: introductory catch-up campaigns*	10-percent points lower than UC1-UC3 combined	10-percent points lower than UC1-UC3 combined	Only used in sensitivity analyses where the TCV vial is not introduced before MAPs become available. Sources: [22] (see Supplementary File 1)
*Use case 5: military*	10%–50% of military population	10%–50% of military population	The size of the military population is estimated to be between 0.5% and 5%, depending on the country [32] (see Appendix A-5). We assumed no change in coverage between TCV vial and MAPs for the military population. Sources: [22] (see Supplementary File 1)
*Use case 6: travelers*	2%–5% of the traveler population	2%–5% of the traveler population	The traveler population is estimated to be 1% of the total population. We assumed no change in coverage between TCV vial and MAPs. Sources: [22] (see Supplementary File 1)
***Delivery cost per dose***			
*Use case 1: fixed post*	Mean $0.43; range $0.21–$2.53	Base profile: mean $0.88; range $0.59–$2.85 Optimistic: mean $0.25; range $0.08–$2.19 Pessimistic: mean $0.96; range $0.59–$3.23	Sources: VTIA analysis for delivery cost estimates (see Supplementary File 1)
*Use case 2: outreach*	Mean $0.52; range $0.16–$1.97	Base profile: mean $1.02; range $0.34–$3.06 Optimistic: mean $0.51; range $0.10–$2.00 Pessimistic: mean $1.02; range $0.59–$3.23	Sources: VTIA analysis for delivery cost estimates (see Supplementary File 1)
*Use case 3: mobile*	Mean: $1.16; range $0.34–$4.25	Base profile: Mean $1.40; range $0.63–$4.17 Optimistic: Mean $0.80; range $0.11–$3.61 Pessimistic: Mean $1.40; range $0.63–$4.17	Sources: VTIA analysis for delivery cost estimates (see Supplementary File 1)
*Use case 4: introductory catch-up campaigns*	$0	Base profile: Mean $0.15; range $0–$3.97 Optimistic: Mean $0.10; range $0–$3.42 Pessimistic: Mean $0.15; range $0–$3.98	Only used in sensitivity analyses where the TCV vial is not introduced before MAPs become available. Sources: VTIA analysis for delivery cost estimates (see Supplementary File 1)
*Use case 5: military*	$0.30	$0.50	Sources: VTIA analysis for delivery cost estimates (see Supplementary File 1)
*Use case 6: travelers*	$0.30	$0.50	Sources: VTIA analysis for delivery cost estimates (see Supplementary File 1)
***Vaccine protection***			
*Efficacy*	85%	85%	We assumed non-inferiority of MAPs compared to the TCV-N&S (see Appendix A-3).
*Duration*	10 years	10 years	
***Product wastage***			
*Vaccine*	11%	1%	Based on revised WHO global indicative wastage rates. Source: [22]
*Auto-disable syringes*	10%	Not applicable	
*Safety boxes*	10%	10%	
***Cost-effectiveness parameters***			
*Time horizon*	20 years (2033–2052)	20 years (2033–2052)	Minimum time frame required to observe impacts on health outcomes and transmission trends.
*Discount rate*	3%	3%	Standardized assumption for the economic analysis of health interventions Source: WHO-CHOICE [33].

**Abbreviations**: **CHOICE**, CHOosing Interventions that are Cost-Effective; **DHS**, demographic and health survey; **MAPs**, microarray patches; **MCV**, measles-containing vaccine; **MICS**, multiple indicator cluster survey; **N&S**, needle and syringe; **RI**, routine immunization; **TCV**, typhoid conjugate vaccine; **VTIA**, vaccine-targeted immunization area; **WHO**, World Health Organization.

Although the TCV-N&S presentation is currently introduced in only five Gavi-eligible countries and two upper-middle-income countries (see Appendix A-4), in this analysis, we assumed that its uptake will increase in the coming years and that it will be introduced in all low- and middle-income countries by 2032, preceding the introduction of TCV-MAPs. Secondly, we assumed TCV-N&S vaccine coverage levels to remain constant over the time horizon and equivalent to the coverage levels of the first MCV1, administered at 9 months of age, given the scarcity of data on TCV coverage and the recent stagnation of MCV1 coverage in many countries [30]. Vaccine coverage data for each country were obtained from the DHS and MICS. Thirdly, we assumed that TCV-N&S delivery occurs via routine and campaign strategies, with campaigns implemented only if there is a need to introduce TCV, and not if a previous TCV-containing product is already in use (e.g., TCV-N&S before TCV-MAPs); other delivery strategies, such as routine use only, are considered in the sensitivity analysis. For adult vaccine recipients (travelers, who are <1 % of the adult population, and military personnel, who account for 0.5–5 % of the population), we assumed that there is no increase in coverage but that there would be a switch due to increased convenience (see Appendix A − 5), as outlined in a previous publication [22].

For TCV-MAPs, we based our assumptions on those of Soble et al. [22]. We assumed that the proportion of the population reached by each delivery mechanism (or use case) remains unchanged after the introduction of TCV-MAP in each country (see Fig. A 11 in Appendix A-6). Any increase in overall coverage is due solely to the increased coverage (of 20 %) for previously unvaccinated populations, such as hard-to-reach groups and those who missed vaccination opportunities, as illustrated for one example country in Appendix A-7. Soble et al. based this assumption on estimates of zero-dose children, i.e., those who have not received any of the recommended vaccines outside conflict settings and who are potentially reachable by overcoming certain programmatic barriers associated with the N&S presentation. In particular, the additional coverage achieved by MAPs among the previously unvaccinated is only attributed to the two lowest wealth quintiles, where data show a disproportionate concentration of unvaccinated individuals (see Fig. A 5 in Appendix A-2). For adult recipients (travelers and military personnel), given the current low vaccination coverage and their small share of the market, we assumed that coverage would not improve with MAPs, but that only the price of the vaccine product used (now being MAPs) would change.

### 2.4 Health outcomes and cost-effectiveness thresholds

The health outcomes of our analysis included the number of typhoid cases, deaths, and disability-adjusted life years (DALYs) averted due to the implementation of TCV-MAPs. We calculated costs in 2021 US dollars and applied a 3 % annual discount rate to future costs and DALYs. The main metric of cost-effectiveness we used is the incremental cost-effectiveness ratio (ICER). A strategy is considered cost-effective compared to another when it yields health benefits at a cost equal to or below a specified willingness-to-pay (WTP) value. This WTP value is commonly referred to as the cost-effectiveness threshold.

We adopted several thresholds, including those proposed by WHO and more recent stringent thresholds [34]. A strategy that prevents a DALY at an additional cost of maximum 0.5 times the GDP per capita of the country is considered “highly cost-effective” (HCE). Between 0.5 and 1 times the GDP per capita is “very cost-effective” (VCE), between 1 and 3 times the GDP per capita is “cost-effective” (CE), and above 3 times the GDP per capita is “not cost-effective” (NCE). If TCV-MAPs prevent an additional DALY at a lower cost compared to TCV-N&S, the new product is considered “cost-saving” (CS). Our approach represents a conservative adaptation of methodologies found in the literature [34,35]. These studies suggest that countries choose a threshold amount equivalent to 0.1 to 1 times their GDP per capita.

### 2.5 Sensitivity analyses

The CEA was conducted based on alternative assumptions outlined in Table 1, column 3. These sensitivity analyses focus on two main categories: 1) product characteristics (price and product profile) and 2) program characteristics. In particular, we vary the coverage of the TCV-N&S by assuming that a) age at routine vaccination at 9 months, assuming MCV1 coverage, would improve, or b) that the coverage is equivalent to that achieved for MCV2 or vaccination at 15 months, the age at which TCV is currently administered in two out of five countries (Table A 4 in Appendix A-4). Differences in coverage per wealth quintile between MCV1 and MCV2 for the countries for which there is data are illustrated in Fig. A 12 in Appendix A-8. Additionally, the impact of the TCV-N&S introduction campaign is assessed by running the analysis assuming that TCV-N&S occurred within the context of a “routine-only” introduction or that it does not occur at all, rather than a “routine and campaign” scenario. Moreover, the market penetration of MAPs varies from 80 % switch to targeted adoption or complete replacement. Lastly, MAPs coverage of unreached populations is assumed to be either 10 % or 30 % instead of 20 %.

### 2.6 Drivers of cost-effectiveness

In addition to the sensitivity analyses, we analyzed the drivers of cost-effectiveness under the default assumptions listed in Table 1. We scaled the ICERs in each country by dividing them by the country's GDP per capita and calculated the Pearson's correlation coefficient between inputs in the model and the log-transformed, scaled ICER. We log-transformed the ICERs because this was better way to explain the relationship with the inputs due to the non-linear nature of our disease model. We considered the inputs in the model that varied by country or district. The following parameters varied by country: mean age of infection without any vaccination, vaccine coverage (MCV1), case-fatality rate, life expectancy mean incidence per 100,000 without vaccination, vaccine coverage (MCV1) within the lowest wealth quintile, improved sanitation coverage, improved sanitation coverage within the lowest wealth quintile, and additional delivery costs of MAPs vs. N&S presentation. Only three sets of parameters varied between regions of any country: coverage of MCV1 by wealth quintile, coverage of improved sanitation by wealth quintile, and poverty—measured as the distribution of individuals in each wealth quintile within the region. Wealth quintiles were based on national wealth rather than district wealth. We examined the direction of the relationship between the scaled, log-transformed ICERs and the scaled predictors (i.e., dividing the values by the standard deviation) using OLS regression and presented the results as a list of drivers ranked by their importance in each country. We also performed a multivariate regression with all the predictors, and then with all the predictors except those with multicollinearity (according to variance inflation factor values of over 10), and then finally with only the predictors that were significant.

### 2.7 Subnational analysis

We conducted a subnational analysis in five countries where we had access to subnational data on key variables: Burkina Faso, Kenya, Malawi, India, and Nepal to assess whether targeting TCV-MAPs to specific locations would enhance the cost-effectiveness of MAPs compared to a national strategy. To do this, we collected data from DHS on district-level prevalence of improved sanitation, vaccine coverage, and the distribution of populations across the five wealth quintiles (where quintiles are based on national-level wealth distribution rather than district-level wealth distribution). The characteristics of typhoid epidemiology, treatment costs, and vaccine costs are detailed in Table A 6 in Appendix A-9.

## 3 Results

### 3.1 Global analysis

#### 3.1.1 Burden

The global impact under different coverage and delivery strategy assumptions before and after the introduction of TCV-MAPs is summarized in Table B 1 in Appendix B-1. Before TCV-MAPs are introduced (2023−2032), TCV-N&S can avert up to 69 million cases compared to no vaccination if rolled out through both routine and campaign strategies. During the time horizon of our analysis (2033–2052), when TCV-MAPs might become available, TCV-N&S could avert 48.9 million cases, assuming no changes in coverage. In the same period, TCV-MAPs could additionally avert an estimated 5.2 million cases due to the assumed coverage improvements (see Table B 1 in Appendix B-1).

**Fig. 1:**
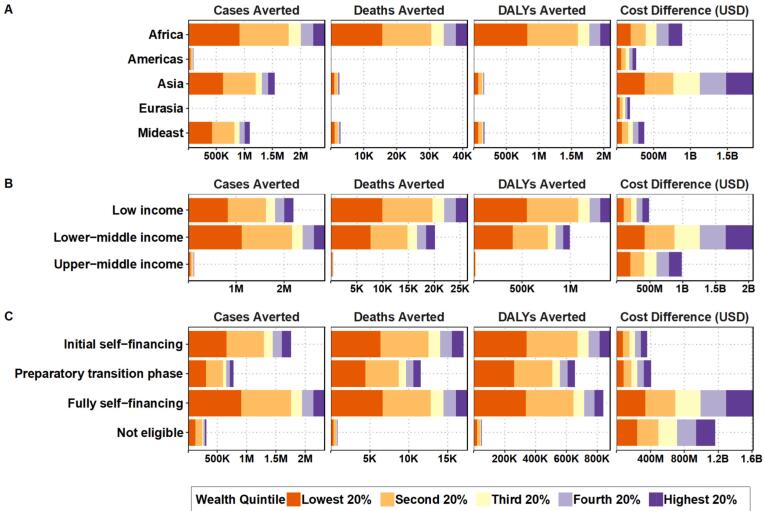
Cases, deaths, and DALYs averted and cost differences per wealth quintile by global region (Panel A), World Bank income classification (Panel B), and Gavi eligibility (Panel C). Abbreviation: DALYs, disability-adjusted life years

Table 2 and Fig. 1 present a detailed overview of cases, deaths, and DALYs averted at the global level and by WHO region. Results from alternative assumptions are shown in Supplemental File 2. A global implementation of TCV-MAPs in all countries could prevent nearly 47,000 deaths and 2.4 million DALYs at an additional cost of $3.5 billion over 20 years. Results for individual countries are presented in Supplemental File 3.

**Table 2:** Total number of typhoid cases, deaths, DALYs and costs with TCV-N&S alone and with TCV-MAPs augmenting TCV-N&S, and the number of typhoid cases, deaths, DALYs averted and additional costs from 2033 to 2052 for 133 countries included in the global analysis. Costs in 2021 US$.

**Outcome**	**With TCV-N&S alone**	**With TCV-MAPs augmenting TCV-N&S**	**Difference between TCV-N&S alone versus with MAPs**	**Percent difference**	**Difference per million population**
***All (133 countries; 6.613B population in 2021)***					
Cases	233,127,507	227,954,337	-5,173,170	−2.2	−782
Deaths	1,554,026	1,507,027	−46,999	−3.0	−7
DALYs	71,590,219	69,158,947	−2,431,272	−3.4	−368
Cost	18,860,709,796	22,402,301,873	3,541,592,077	18.8	535,557
***Africa (45 countries; 1.149B population in 2021)***					
Cases	70,754,931	68,323,829	−2,431,102	−3.4	−2117
Deaths	1,258,848	1,217,355	−41,494	−3.3	−36
DALYs	56,113,130	54,014,040	−2,099,090	−3.7	−1828
Cost	5,492,616,318	6,377,737,708	885,121,390	16.1	770,613
***Americas (26 countries; 626*** ***M population in 2021)***					
Cases	5,091,149	5,001,755	−89,394	−1.8	−143
Deaths	6461	6347	−114	−1.8	> − 1
DALYs	332,868	325,870	−6998	−2.1	−11
Cost	759,003,602	1,020,990,025	261,986,423	34.5	418,603
***Asia (26 countries; 3.709B population in 2021)***					
Cases	124,189,707	122,648,942	−1,540,765	−1.2	−416
Deaths	202,925	200,421	−2504	−1.2	−1
DALYs	10,837,280	10,677,407	−159,873	−1.5	−43
Cost	8,995,947,198	10,839,246,669	1,843,299,471	20.5	497,431
***Eurasia (20 countries; 419*** ***M population in 2021)***					
Cases	2,255,040	2,241,125	−13,915	−0.6	−33
Deaths	4331	4298	−34	−0.8	> − 1
DALYs	217,458	215,438	−2020	−0.9	−5
Cost	512,054,557	692,259,414	180,204,857	35.2	430,177
***Mideast (16 countries; 714*** ***M population in 2021)***					
Cases	30,836,679	29,738,686	−1,097,993	−3.6	−1538
Deaths	81,461	78,607	−2854	−3.5	−4
DALYs	4,089,483	3,926,191	−163,292	−4.0	−229
Cost	3,101,088,121	3,472,068,057	370,979,936	12.0	519,639

**Abbreviations**: **DALYs**, disability-adjusted life years; **MAPs**, microarray patches; **N&S**, needle and syringe; **TCV**, typhoid conjugate vaccine; **WHO**, World Health Organization.

Note: The countries and the populations listed are only those in the analysis. Populations are based on 2021 estimates [36].

Fig. 1 and Table 2 highlight that the majority of the burden would be averted in low- and lower-middle-income countries, especially in sub-Saharan Africa, where 2.4 million cases, over 41,000 deaths, and 2.1 million DALYs would be averterd, at a additonal cost of $885 million. The largest cost increase is in Asia, where 1.5 million cases, approximately 2500 deaths, and 160,000 DALYs could be prevented at an additonal cost of $1.8 billion, due to its larger population and lower typhoid incidence and case-fatality rate (CFR). Generally, most of the burden is averted in the lowest two wealth quintiles within a country, while the costs are more evenly distributed across all wealth quintiles.

#### 3.1.2 Baseline cost-effectiveness analysis

The baseline CEA, assuming a TCV-MAP price of $3 and an 80 % switch to TCV-MAPs, found that MAPs could be cost-effective or cost-saving compared to TCV-N&S in approximately 44 (33.1 %) of the 133 countries, or 14.5 % of the global population. Specifically, TCV-MAPs could be cost-saving in Yemen and highly cost-effective in 11 countries (8.3 % of the countries): Mauritius, Gabon, Guinea, Benin, Botswana, Côte d'Ivoire, Nigeria, Papua New Guinea, Angola, Ethiopia, and the Democratic Republic of the Congo. Additionally, TCV-MAPs could be very cost-effective in 6 countries (4.5 %): South Africa, Republic of Congo, Mauritania, Equatorial Guinea, Liberia, and Madagascar. TCV-MAPs could also be cost-effective in 26 more countries but not cost-effective in the remaining 89 countries. For detailed country-specific information, see Supplemental File 3.

**Fig. 2:**
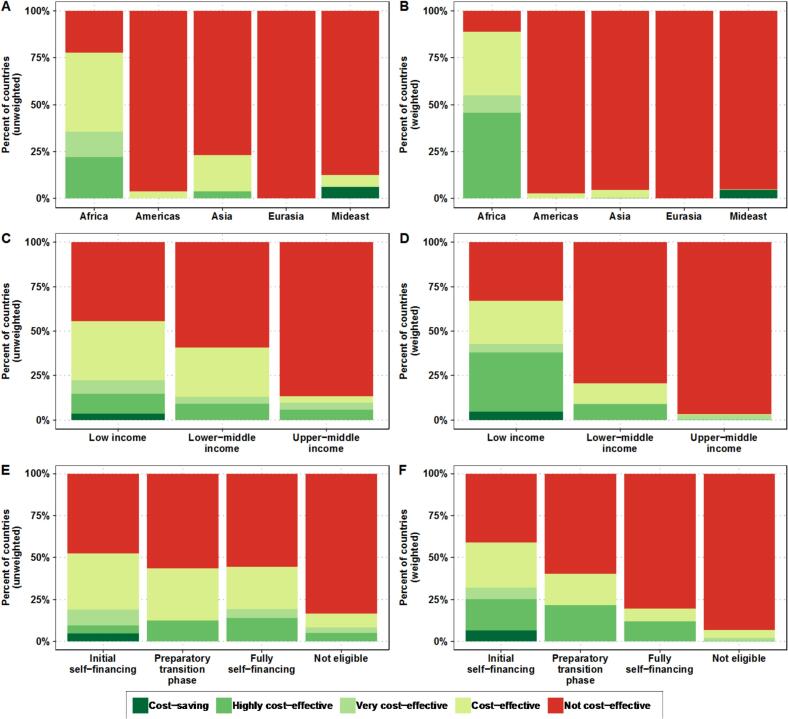
Cost-effectiveness unweighted (left) and weighted by population size (right) for each global region (Panel A and B), income classification (Panel C and D), and Gavi eligibility (Panel E and F). Results are shown for the default assumptions (TCV-MAP coverage of 20 % of unvaccinated individuals, TCV-MAP price of $3.00 per dose, and TCV-MAP market penetration of 80 %). On the left, the unweighted results are shown; that is, a simple percentage of the countries in each cost-effectiveness category shown in the legend. On the right, the weighted results are shown, which represent the percentage of countries weighted by population size; that is, the population across all countries that belongs to countries in each of the cost-effectiveness categories shown in the legend.

TCV-MAPs could be cost-effective in a majority of African countries (78 %), representing about 89 % of the population in this region (Fig. 2). In other regions, TCV-MAPs are generally not cost-effective for the majority of the countries and populations. Additionally, TCV-MAPs are more likely to be cost-effective in low-income countries, followed by lower-middle-income countries, and for approximately half of Gavi-eligible countries.

#### 3.1.3 Sensitivity analyses

##### 3.1.3.1 Product characteristics

**Fig. 3:**
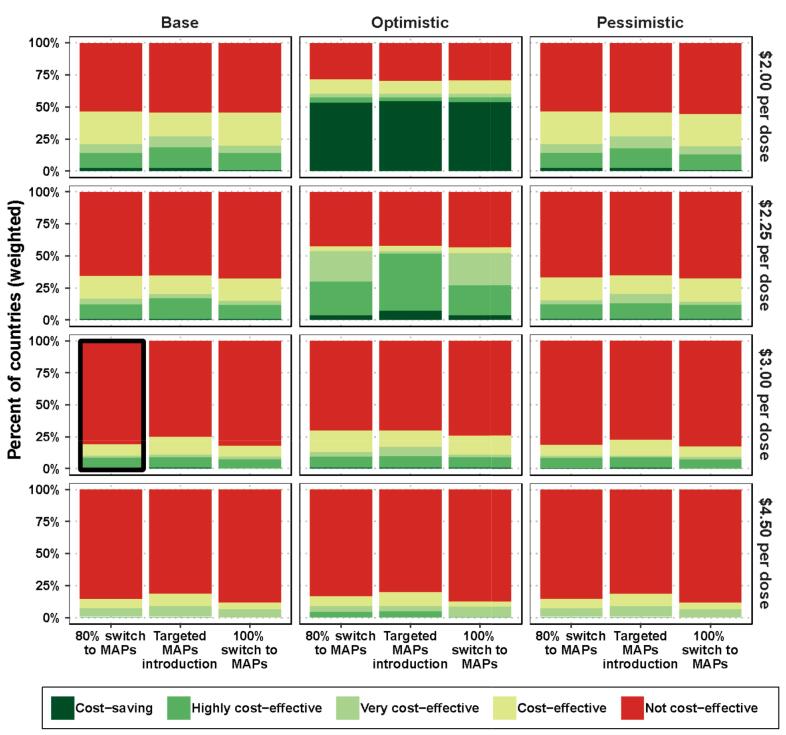
Cost-effectiveness by vaccine price, comparator, and MAP profile, weighted by population size. The column marked with a black box represents the results under our default assumptions. Fig. B 2 in Appendix B-3 shows the unweighted results; that is, a simple percentage of countries in each cost-effectiveness category shown in the legend. Here, we show the percentage of countries weighted by population size; that is, the population across all countries within each of the cost-effectiveness categories shown in the legend. Abbreviation: MAPs, microarray patches

The sensitivity analysis indicates that at a price of $3 per dose, TCV-MAPs are likely to be cost-effective for 19 % of the global target population in 33 % of countries across the MAPs and TCV-N&S mix scenarios (see [Insert Fig. 3 for population-weighted results; see Fig. B 2 in Appendix B-3 for unweighted results). However, at a price of $2.25 per dose, TCV-MAPs are likely to be cost-effective for up to 59 % of countries or 34 % of the target populations. Among different MAPs and N&S mix scenarios, TCV-MAPs are likely to be slightly less cost-effective when MAPs completely replace vials, but MAPs might show advantages in a targeted approach.

At lower MAP prices, an optimistic MAP profile would lead to cost savings for over 38 % of countries or 72 % of the global population driven mostly by the lower cold-chain volume. With the base or pessimistic MAP profiles, cost savings are not achieved by TCV-MAPs, although the base profile could be cost-effective for 24 % of the countries or 14 % of the global target population at a price of $4.50 per dose and almost 62 % of countries or 47 % of the target population at the lowest price of $2 per dose.

##### 3.1.3.2 Program characteristics

The uncertainty around key parameters, such as the coverage level of TCV-MAPs, the rollout strategy of TCV-N&S, and TCV-N&S (MCV1) vaccine coverage, and their impact on cost-effectiveness results is shown in Fig. B 3, Fig. B 4, and Fig. B 5 in Appendix B-4, respectively.

We observe that increased coverage assumptions for TCV-MAPs systematically increase the proportion of populations and countries for which MAPs could be cost-effective at $2.25 per dose and $3.00 per dose for the base and optimistic profiles. The value proposition of TCV-MAPs does not change substantially if TCV-N&S are only introduced in routine settings. However, if TCV in vial presentation is not introduced, TCV-MAPs would become cost-effective compared to no vaccination in approximately 75 % of countries and 85 % of the populations at $2.25 per dose and $3.00 per dose for the base and optimistic profiles. Higher baseline levels of MCV1 coverage (and thus higher baseline TCV-N&S coverage given we assumed TCV N&S coverage is the same as for MCV1) reduce the proportion of populations where TCV-MAPs could be cost-effective. Conversely, since MCV2 coverage levels are generally lower than those for MCV1, the proportion of countries for which TCV-MAPs could be cost-effective is greater if we assume baseline TCV-N&S coverage is the same as MCV2 coverage levels.

#### 3.1.4 Inequality

**Fig. 4:**
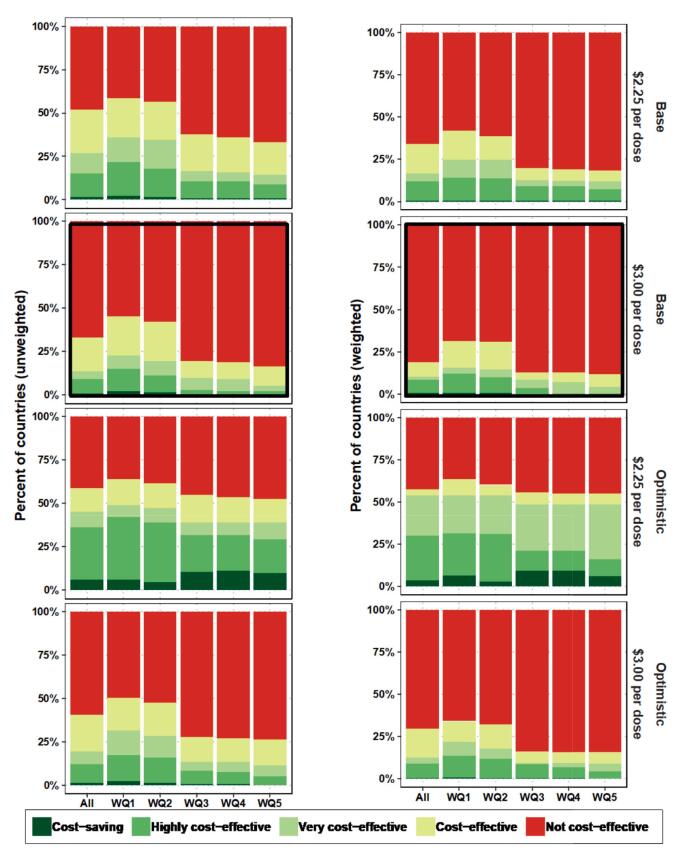
Cost-effectiveness by wealth quintile, vaccine price, and MAP profile, unweighted (left) and weighted by population size (right). On the left, the unweighted results are shown; that is, a simple percentage of the countries in each cost-effectiveness category shown in the legend. On the right, the weighted results are shown, representing the percentage of countries weighted by population size; that is, the population across all countries within each of the cost-effectiveness categories shown in the legend. The panels marked with black boxes represent the results of our default assumptions.

In the baseline results, we observed that the lowest wealth quintiles experience the largest reduction in typhoid burden from a TCV-MAPs introduction, assuming additional vaccine coverage is allocated to these groups. Therefore, TCV-MAPs are most likely to be cost-effective for lower wealth quintiles, although indirect effects of higher vaccination show benefits in some populations of higher wealth quintiles ([Insert Fig. 4). The cost savings or proportion of cost-effectiveness increase with a more price-competitive product and an “optimistic” product profile. Another way to examine the impact of TCV-MAPs on inequality is to compare the reduction in inequality before and after TCV-MAPs deployment, as shown in Appendix B-5. Fig. B 6 shows that the reduction in inequality is likely to be higher when existing inequality between wealth groups is higher, and this pattern holds for both excess cases and DALYs. In other words, TCV-MAPs will produce more benefit where inequalities are higher, but at our assumed 20 % coverage of the unvaccinated population, this translates to a modest reduction in inequality of up to only 10 % to 12 % in terms of cases and DALYs. Fig. B 7 expands upon this and shows that the ICERs are lower—thus, TCV-MAPs are more cost-effective—when the reduction in inequality is higher.

#### 3.1.5 Drivers of cost-effectiveness

**Fig. 5:**
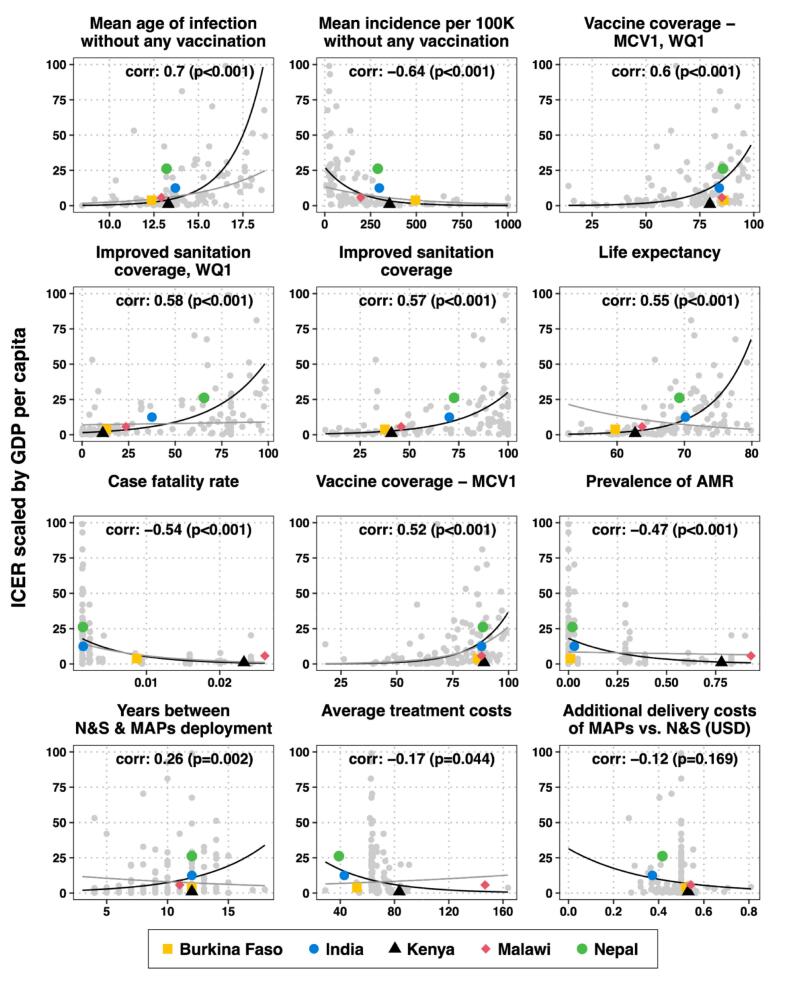
Drivers of cost-effectiveness, ordered by strength of correlation with the ICER scaled by GDP per capita. The gray dots represent each country in the analysis, with the five countries in the deep dive marked in colored dots. The black lines represent univariate correlations between each input and the scaled ICERs, and the gray lines represent the coefficient of correlation between input and the scaled ICERs after adjusting for other inputs in a multivariate regression. Inputs that do not have a gray line were omitted from the multivariate model due to collinearity with other inputs. The correlations are examined using log-linear regression, which fit better than a linear regression due to the non-linear nature of infection dynamics. Abbreviations: AMR, antimicrobial resistance; GDP, gross domestic product; ICER, incremental cost-effectiveness ratio; MAPs, microarray patches; MCV, measles-containing vaccine; N&S, needle and syringe; USD, united states dollar; WQ, wealth quintile.

Among the 12 different drivers of cost-effectiveness explored, 11 drivers are statistically significant at a 5 % significance level. The age of infection, pre-vaccine incidence, and vaccine coverage among the poorest wealth quintile have the highest correlation with cost-effectiveness (Fig. 5 and Fig. B-8 in Appendix B-6). Due to increased DALYs averted, a lower mean age of infection favors adoption of MAPs, as does lower vaccine coverage, since TCV-MAPs should compensate for shortfalls in coverage under the N&S presentation. Incidence has the expected relationship—higher incidence favors adoption of MAPs—and is statistically significant, both in univariate and multivariate analyses. While overall vaccine coverage has a significant correlation with ICERs, vaccine coverage in the poorest wealth quintile has an even stronger and statistically significant correlation; both of these variables are collinear, as expected, and the variable inflation factor (VIF) determined that we should remove the vaccine coverage in the poorest quintile from the multivariate model. Conversely, a higher typhoid CFR or prevalence of AMR favors the adoption of TCV-MAPs due to its impact on DALYs in terms of years of life lost.

Some inputs have relationships to ICERs that are reversed upon adjustment of other factors: years between N&S and MAPs deployment as well as average treatment costs have mild correlations with ICERs that are reversed upon adjustment of other factors. The analysis showed that the higher the life-expectancy, the higher the ICER, but after adjusting for covariates, the relationship became negative; a longer life expectancy would lead to more DALYs being lost due to a fatality, therefore favoring adoption of MAPs. In contrast, sanitation had no significant influence, both overall and in the poorest wealth quintile when we considered the explanatory power of other variables. Higher delivery costs for TCV-MAPs compared to TCV-N&S resulted in lower ICERs, but it was not statistically significant in univariate or multivariate analyses.

### 3.2 Subnational analysis

#### 3.2.1 Baseline cost-effectiveness analysis

Table 3 provides an overview of the burden averted and the associated costs for both national and subnational implementations of TCV-MAPs for the five countries selected for the subnational analysis. The scaled ICERs indicate that, under default assumptions, a national implementation of TCV-MAPs would only be cost-effective in Kenya, while it would not be cost-effective in the other four countries evaluated. At a lower price of $2.25, TCV-MAPs would also be cost-effective in Burkina Faso. However, with both a reduced price per dose of $2.25 and an optimistic product profile, a national introduction of TCV-MAPs could be cost-effective in four out of the five countries—Burkina Faso, India, Kenya, and Malawi. On the other hand, MAPs would not be cost-effective in Nepal for any of the scenarios evaluated.

**Table 3:** Overview of health outcomes and CEA results for national versus subnational introduction of TCV-MAPs in five selected countries.

	Burkina Faso	India	Kenya	Malawi	Nepal
*National introduction (base MAP profile, $3*)					
Cases averted	19,743	571,400	37,690	6941	9393
Deaths averted	171	782	878	181	13
DALYS averted (discounted)	3404	17,714	17,384	3546	287
Additional cost (discounted)	14.2 M	662.2 M	45.8 M	14.5 M	16.4 M
Cost-effectiveness (ICER scaled by GDP per capita)	5.06 (Not cost-effective)	15.5 (Not cost-effective)	1.26 (Cost-effective)	6.35 (Not cost-effective)	42.9 (Not cost-effective)

*Subnational introduction (base MAP profile, $3)*					
Cases averted	3194	86,864	35,766	178	0
Deaths averted	28	118	833	5	0
DALYs averted (discounted)	548	2655	15,527	87	0
Additional cost (discounted)	434,948	10.4 M	36.6 M	100,628	0
Cost-effectiveness (ICER scaled by GDP per capita)	0.95	1.62	1.05	1.80	0
# districts where MAPs could be cost-effective	1 (8 %)	63 (9 %)	38 (81 %)	2 (6 %)	0 (0 %)

*Sensitivity analysis - national (ICERs scaled by GDP* per capita*)*					
Optimistic, $3	2.98	8.95	0.74	3.70	28.07
Base, $2.25	2.73	7.65	0.67	3.40	20.84
Optimistic, $2.25	0.65	1.09	0.16	0.75	6.04

*Sensitivity analysis - subnational (# districts where MAPs could be cost-effective*)					
Optimistic, $3	6 (46 %)	103 (15 %)	42 (89 %)	11 (34 %)	2 (3 %)
Base, $2.25	6 (46 %)	138 (20 %)	45 (96 %)	14 (44 %)	3 (4 %)
Optimistic, $2.25	13 (100 %)	488 (69 %)	47 (100 %)	29 (91 %)	10 (13 %)
# districts (total)	13	707	47	32	77

**Abbreviations**: **CEA**, cost-effectiveness analysis; **DALY**, disability-adjusted life year; **GDP**, gross domestic product; **ICER**, incremental cost-effectiveness ratio; **MAPs**, microarray patches; **TCV**, typhoid conjugate vaccine.

Thresholds for determining cost-effectiveness when ICER is scaled by GDP: A strategy that prevents a DALY at an additional cost of maximum 0.5 times the GDP per capita of the country is considered “highly cost-effective” (HCE). Between 0.5- and 1-times GDP per capita is “very cost-effective” (VCE), between 1- and 3-times GDP per capita is “cost-effective” (CE), and above 3 times GDP per capita is “not cost-effective” (NCE).

Since disparities in typhoid risk factors are not only observed between countries but also within countries, subnational regions (e.g., districts or counties) could still benefit from the introduction of TCV-MAPs without incurring the full costs of a national implementation. If TCV-MAPs were introduced only in districts where they would be cost-effective, in Burkina Faso, this would mean introducing TCV-MAPs in only 1 out of 13 districts in the country. The introduction of TCV-MAPs in that one district could reduce the total number of typhoid cases by 15 % (based on the national caseload) and at 3 % of the cost of a national introduction, assuming a base MAP profile priced at $3 per dose. As expected, improvements in cost-effectiveness can be achieved once we consider a lower price and an optimistic TCV-MAP profile. Similarly, in India, a subnational strategy could be cost-effective when TCV-MAPs are introduced in 63 out of 707 districts, reducing cases by 15 % at just 1.6 % of the national cost. In Malawi, subnational implementation of TCV-MAPs could avert 3 % of cases at less than 1 % of the national cost. However, in Kenya, a subnational strategy offers little additional value since national implementation is already cost-effective under default assumptions. In Nepal, segmentation by district would not change the value proposition for TCV-MAPs, as it remains not cost-effective at subnational levels. Maps showing cost-effectiveness results for all five countries are shown in Fig. C 1 through Fig. C 5 in Appendix C-1.

#### 3.2.2 Sensitivity analysis

**Fig. 6:**
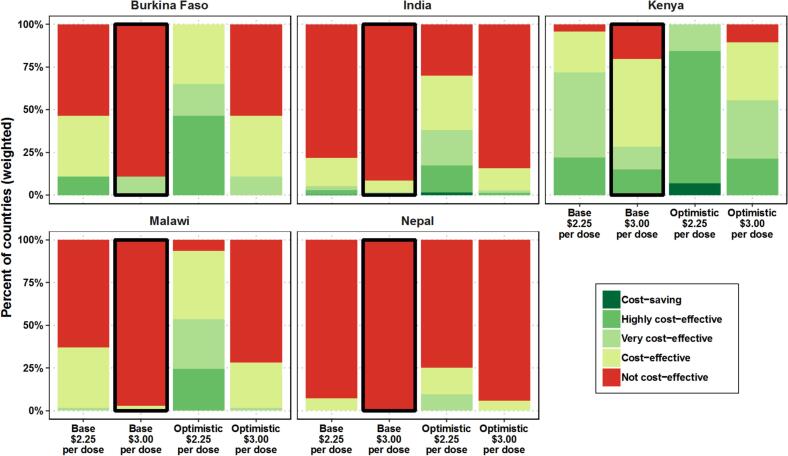
Impact of vaccine price and MAP profile on the cost-effectiveness results for all countries included in the subnational analysis. The results represent the percentage of districts within each country, weighted by population size; that is, the population across all districts within a country that falls into each of the cost-effectiveness categories shown in the legend. The columns marked with a black box represent the results based on our default assumptions.

Consistent with our results presented above, the sensitivity analysis indicates that in countries where subnational implementation might be cost-effective, the price and profile of the TCV-MAP significantly impact the outcomes (Fig. 6). In Burkina Faso, for instance, changing the price from $3 to $2.25 or having a more optimal MAP profile (e.g. smaller volume, faster administration time, as defined in Table 1), can increase the percentage of districts and the population where TCV-MAPs are cost-effective from less than 25 % to over 60 %. In India, the price only has a substantial impact when an optimistic MAP profile is considered. Conversely, in Kenya and Nepal, the cost-effectiveness results are minimally influenced by either the price or the MAP profile.

#### 3.2.3 Drivers of cost-effectiveness

The drivers of cost-effectiveness for the subnational analysis are detailed in Table C 1 in Appendix C-2. In summary, we observe that MCV1 coverage shows a positive and significant association with the ICER, both independently and jointly with other drivers, contributing to the variation in subnational ICERs. On the other hand, sanitation and poverty do not exhibit a consistent relationship, either in direction or significance, across the five countries.

## 4 Discussion

This study aimed to elucidate the potential socioeconomic and public health impacts of the introduction and use of a novel vaccine presentation for typhoid, TCV-MAP, compared to TCV-N&S. The results indicate that TCV-MAPs are more likely to be cost-effective in sub-Saharan Africa, due to the larger proportion of unvaccinated populations and the higher likelihood of intestinal perforations and mortality. However, TCV-MAPs are unlikely to be cost-effective for most other countries and populations, whether adopted alongside TCV-N&S or as a full replacement of TCV-N&S, compared to vaccination with TCV-N&S alone. Previous evaluations of other MAP products (MR, hepatitis B virus [HBV]) have shown that MAP presentations could be a cost-effective way to improve vaccination coverage [37,38]. Unlike chronic HBV, most cases of typhoid are acute and do not result in long-term disability, which might explain why MAPs have a greater impact on HBV burden over a lifetime, as they can prevent lifelong treatment and costs. On the other hand, similar to Fu et al. [34], who evaluated the MR-MAP, our study showed that lower prices for TCV-MAPs, combined with lower initial TCV coverage, improve the value proposition of TCV-MAPs. Additionally, TCV-MAPs are more likely to be cost-effective in countries with a higher typhoid burden.

As expected, our analysis showed that TCV-MAPs would have the greatest impact in the wealth quintiles where it increases coverage the most, particularly the two lowest wealth quintiles, which typically have lower coverage and higher disease burdens. Given the dynamic nature of the model, the analysis by wealth quintile also demonstrates that the indirect effects of MAPs might provide sufficient benefits to higher wealth quintiles, making them cost-effective for these groups in some countries. This finding is consistent with a study by Verguet et al., which reported that public financing of rotavirus vaccination would primarily benefit the poor, but potential indirect benefits of vaccination would increase benefits across all income groups [39]. Furthermore, TCV-MAPs are likely to provide the greatest benefit in populations with the highest inequality in burden. Although the relationship is somewhat noisy due to various factors in the simulation, reducing inequality positively influences the ICER.

While our analysis indicates that the greatest health impact from MAPs arises from routine immunization, it also included a product switch among travelers and military personnel, as country consultations suggested a willingness to use TCV-MAPs among these groups [22]. However, the consultations did not foresee any increase in coverage among these groups, which currently have low coverage and represent a small portion of the potential TCV-MAP market. Therefore, simulating a product change for these groups in our model resulted in a higher product price but no impact on disease transmission. The increase in cost at the population level would likely be minimal, as these populations are small and represent a minor portion of the annual TCV-MAP recipients.

The modest reductions in inequality (10–12 % reduction in the differences between the poorest and richest quintile) that we estimated could be achieved by a TCV-MAP highlight the need to address inequalities holistically, including in disease exposure prevention (sanitation) in addition to prophylaxis. Ultimately, inequalities in access to improved sanitation are more severe than the inequalities in access to vaccination. Since the vaccine does not confer 100 % immunity or lifelong protection, the risk of infection persists even with high vaccine coverage.

Our subnational analysis indicated that a subnational introduction of TCV-MAPs might be valuable in cases where a national introduction is not cost-effective. A subnational implementation strategy could offer better value for money in regions with high typhoid mortality and significant heterogeneity in sanitation and vaccine coverage across regions. Health impact could be achieved with a subnational introduction at a lower cost than a national introduction. However, our analysis did not explore the implementation process for such a subnational strategy.

The most significant drivers of variation in ICERs in the global analysis are mean age of infection, incidence of typhoid before any vaccination, and vaccine coverage. Moreover, our analysis found that TCV-MAP price is a critical driver of cost-effectiveness. As the product moves from clinical development to the market over the next few years, optimizing production costs and reducing the product price will be crucial to ensure it is offered at a viable and acceptable price. In addition, factors affecting delivery costs, such as cold chain volume and wear time, though less influential than the price, will be important to optimize in order to further increase the likelihood of uptake, acceptance, and improve the value proposition of this new delivery technology.

It is noteworthy that the TCV-N&S is approved for use in a CTC at the last mile, just before vaccine administration. Therefore, a significantly improved thermostability profile for TCV-MAPs, as modeled for at least two months, would be required for this thermostability feature to bring programmatic value and extend reach and coverage beyond what can be achieved with the vial presentation [40]. Further improvements in thermostability for TCV-MAPs could lead to reduced delivery costs and increased coverage, stemming from simplified storage and transportation at various levels of the supply chain, administration by lower-skilled health professionals, and increased uptake.

### 4.1 Limitations

Our analysis has several limitations. A key assumption in our analysis was that MAPs could be able to increase coverage among those currently unreached by TCV N&S. Studies have shown that using innovative needle-free delivery technologies enabled increased coverage of inactivated poliovirus vaccines compared to what was achieved with N&S [41,42]. There is not yet such real-world evidence for MAPs, but as pilot studies are conducted during implementation of MAP products such as MR-MAPs, this assumption can be validated for MAPs.

There are also several limitations regarding the data. First, due to a lack of country-specific mortality data for most countries included in the analysis, mortality was estimated at the continental level and extrapolated for countries where data were missing. This approach may have led to an overestimation of the cost-effectiveness of TCV-MAPs in countries with lower-than-average mortality and an underestimation in those with higher-than-average mortality. Second, since only five Gavi-eligible countries had implemented TCV-N&S as of 2023, we did not have direct TCV coverage data and instead used MCV1 coverage, which targets the same population for routine immunization as TCV. While we assumed that TCV coverage would match that of MCV1, two of the five countries that have implemented TCV did so during the MCV2 visit (at 15 months of age). Since MCV2 typically has lower coverage, this conservative assumption could make MAPs more cost-effective in more countries. Third, typhoid incidence data were derived from two models that rely on fewer than 40 studies of blood-culture-confirmed, population-based estimates. Given that typhoid is a relatively understudied disease, many other estimates are based on limited or outdated studies. For instance, the most reliable estimates of the probability of being a chronic carrier date back to 1943 [26]. Additionally, there is limited evidence on how AMR status influences care-seeking behavior, hospitalization, costs, and mortality. To maintain consistency, we aligned our assumptions with those of a previous cost-effectiveness study on TCV vials [25].

Additionally, within the subnational analysis, we retrieved district- or county-level data from DHS or MICS for four out of the five countries. However, for Burkina Faso, only regional data were accessible, potentially masking district-specific benefits within broader regional patterns Furthermore, delivery costs were not disaggregated by subnational region in any of the five countries due to data limitations. In addition, due to data limitations, our analysis considered only one risk factor: the prevalence of basic sanitation, excluding the prevalence of improved water sources. This was due to limited differentiation between wealth quintiles in access to improved water. Additionally, health care access was not included as a parameter in the analysis, despite its potentially substantial impact on disease outcomes, particularly in lower wealth quintiles. From the existing data, it was not possible to disentangle the proportion of care specific to typhoid from that related to other febrile diseases. We also did not conduct a probabilistic sensitivity analysis (PSA). For evaluating the features of a TCV-MAP product, a PSA would offer limited insights because our analysis is designed to inform global stakeholders rather than those in any specific country. We focused on the impact of product characteristics on cost-effectiveness, some of which are qualitative and therefore require a scenario analysis, rather than an uncertainty analysis. Lastly, our analysis serves as an early-stage value proposition, relying on assumptions or data from general literature, meaning key assumptions, such as the coverage gain that could be achieved with MAPs, are not validated by real-world data. Furthermore, the MAP profile assumptions and price are unknown. As better data from implementation studies become available and clarity on the technically feasible TCV-MAP profile is achieved, the value proposition will need to be refined to factor in these new data.

### 4.2 Conclusion

This evaluation demonstrates that TCV-MAPs are likely to be cost-effective in countries within sub-Saharan Africa due to the higher disease burden but are less likely to be cost-effective in most countries outside this region. Additionally, a regional or subnational introduction could enhance the value proposition of TCV-MAPs in countries where a national introduction may not be cost-effective due to varying subnational disease burdens. Furthermore, the use of TCV-MAPs could improve health equity in vaccination across income groups within countries. Our analysis also found that the TCV-MAP price is an important driver of cost-effectiveness. This study is the first global cost-effectiveness analysis of TCV-MAPs and thus supports the ongoing research and development of this novel technology, which aims to generate evidence during the development phase for decision-makers. As better data and more insights into potential TCV-MAP price ranges and product characteristics become available, the value proposition for TCV-MAPs should be reassessed.

## Source of support

This work was funded by the Wellcome Trust through award number 224004/Z/21/Z to Gavi.

## Data access

The code for this analysis is available at: https://github.com/Marina-Antillon/TCV_MAPs_CEA

All the data used for the analysis are available in the Supplementary files.

## CRediT authorship contribution statement

**Marina Antillon:** Writing – original draft, Validation, Methodology, Formal analysis, Data curation, Conceptualization. **Anna Verjans:** Writing – review & editing, Validation, Data curation. **Fayad El Sheikh:** Writing – review & editing, Validation, Project administration, Methodology, Conceptualization. **Tiziana Scarna:** Writing – review & editing, Validation, Funding acquisition, Conceptualization. **Mercy Mvundura:** Writing – review & editing, Supervision, Methodology, Data curation, Conceptualization.

## Declaration of competing interest

The authors declare that they have no known competing financial interests or personal relationships that could have appeared to influence the work reported in this paper.

## Data Availability

The data used in the analysis have been submitted at Supplemenraty files. We have also provided a link to the analysis code on Github

## Appendix A Supplementary data

Supplementary material 1: Country-specific data used in the analyses. Supplementary material 1

Supplementary material 2: Estimates of the cases, deaths, and DALYs averted and incremental costs at the global level with alternative model assumptions. Supplementary material 2

Supplementary material 3: Estimated cases, deaths, and DALYs averted and incremental cost-effectiveness ratios by country. Supplementary material 3

Supplementary material 4: Consolidated Health Economic Evaluation Reporting Standards (CHEERS) checklist. Supplementary material 4
