# Supplementary material for: Microarray patch vaccines for typhoid conjugate vaccines: A global cost-effectiveness analysis
Source: Vaccine. 2025 Apr 19;53:None. doi: 10.1016/j.vaccine.2025.127055 (PMC12042820; doi:10.1016/j.vaccine.2025.127055)
Supplement: Supplementary material 1: Country-specific data used in the analyses. [file mmc1.docx]

**Appendix A**

**Methods**

**Appendix A-1:** TCV presentation profiles

Table A‑1. Complete overview of the different TCV-MAP profiles in comparison to the TCV-N&S.

*These assumptions are based on the target product profiles for other vaccine MAPs (human papillomavirus, MR, and rabies), publicly available information from MAP developers, and characteristics of prequalified TCVs.*

| **Attribute** | **TCV-N&S**  **5-dose vial** | **Baseline TCV-MAP profile**  (Based on existing MAP attributes) | **TCV-MAP 1**  **(“Pessimistic”)** | **TCV-MAP 4**  **(“Optimistic”)** |
| --- | --- | --- | --- | --- |
| **Human resource time for administration**  (including wear time) | **17.25 seconds** | **70 seconds**  Delivery time is 50% of one TCV-N&S injection. | **5 minutes**  Delivery time should be comparable to one TCV-N&S injection. | **15 seconds**  Delivery time should be shorter than one TCV-N&S injection. |
| **Wear time** | Not applicable | **60 seconds** | **Up to 5 minutes** | **Under 10 seconds** |
| **Storage volume**  (cold chain volume) | **2.9 cm^3^/dose** | **20cm^3^** | **20 cm^3^** | **5cm^3^** |
| **Thermostability** | Storage at +2°C to +8°C.  Opened vial should be used within 28 days when stored at +2°C to +8°C.  **In CTC:** 7 days at 40°C \| 3 days at 55°C. | Stability profiles equivalent to TCV-N&S. | | Stability profiles should have **enhanced thermostability** (i.e., use under CTC 40°C conditions for at least 2 months). |
| **Volume of injection syringe** | 42.83 cm^3^ | 0 | | |

Abbreviations: CTC, controlled temperature chain; MAP, microarray patch; TCV-MAP, typhoid conjugate vaccine with microarray patch; TCV-N&S, typhoid conjugate vaccine with needle and syringe.

**Appendix A-2:** Transmission and treatment model

The model is a compilation of two models— a probability tree of disease outcomes and a dynamic SIR model of transmission. Figure A-1 shows the decision model, or the overall simulation scheme. For each strategy, the SIR (dynamic) model is run while taking into account only N&S vaccination (Strategy 1) or N&S followed by some MAP vaccination sometime later (Strategy 2). The dynamic disease transmission model is shown in Figure A-2. For the cases that get typhoid each year, then a probability-tree model (Figure A-3) is run to determine the outcomes (recovery or death) and count the DALYs incurred.

Abbreviations: SIR, susceptible-infected-recovered; TCV-MAP, typhoid conjugate vaccine with microarray patch; TCV-N&S, typhoid conjugate vaccine with needle and syringe.

Figure A‑1. Decision model comparing TCV-N&S only versus TCV-MAPs alongside TCV-N&S.


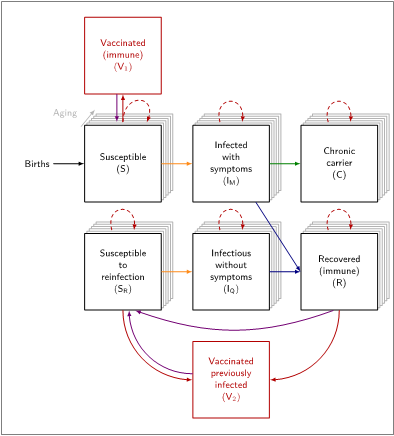

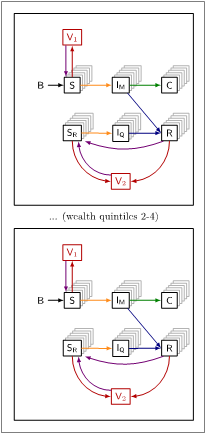


Figure A‑2. Dynamic disease-transmission model.

Transmission was modeled via the SIR model stratified by age and wealth quintile.

Abbreviations: AMR, antimicrobial resistance; IP, intestinal perforation.

Figure A‑3. Probability tree of disease outcomes.

*The final outcomes of disease (recovery or death) are determined by a set of conditional probabilities about the disease. These are parameterized according to findings in the literature. While we discussed the inclusion of nodes to represent the potential of vaccines to protect against severe disease, we decided against this inclusion due to a lack of evidence about the influence of vaccination on severity.*

The SIR model features two susceptible compartments, one for people who are completely immunogenically naïve (S_1_, who have never been infected before) and another for people who have been infected before but whose infection-derived immunity has waned (S_2_). There are two infection compartments (I_M_ and I_Q_): one for primary infections (I_M_), assumed to be the only infections that are observed; and another for secondary and later infections (I_Q_), assumed to be quiet (subclinical) but to contribute to the force of infection, nonetheless. Furthermore, there is a permanent chronic carrier status and temporary immune status (*C*, *R*) and two compartments for temporary vaccine-derived immunity (*V*_1_, *V*_2_). Unlike in other iterations of the model [1-5], there is no water compartment, as water transmission is rarely identifiable from person-to-person transmission (from *I*_M_, *I*_Q_, and *C*) and no interventions listed in these analyses target water-mediated transmission directly. The version of the model without water transmission has already been used (for fitting, validation, and simulation) for cost-effectiveness analyses of TCV vaccine in previously published studies [6, 7]. Mathematically, the flow of individuals between compartments is formalized by a system of ordinary differential equations, as shown in Table A-2.

Table A‑2. A breakdown of the system of ordinary systems of equations that formalizes the dynamic transmission model in Figure A-2.

| **Compartment** | **Abbr.** | **Without vaccination** | **With vaccination**  **(vaccination components in red)** |
| --- | --- | --- | --- |
| **Susceptibles (to first-time infection)** | *S* | $\frac{dS}{dt}=bN-\lambda S$ | $\frac{dS}{dt}=bN-\lambda S-\kappa\nu S+\omega_{V}V_{1}$ |
| **Infected with symptoms** | *I_M_* | $\frac{dI_{M}}{dt}=\lambda S-\delta I_{M}$ | $\frac{dI_{M}}{dt}=\lambda S-\delta I_{M}$ |
| **Recovered** | *R* | $\frac{dR}{dt}=\delta\left( 1-\theta-\alpha\right)I_{M}+{\delta I}_{Q}-\omega R$ | $\frac{dR}{dt}=\delta\left( 1-\theta-\alpha\right)I_{M}+{\delta I}_{Q}-\omega R-\kappa\nu R$ |
| **Chronic carriers** | *C* | $\frac{dC}{dt}=\delta\theta I_{M}$ | $\frac{dC}{dt}=\delta\theta I_{M}$ |
| **Susceptibles (to reinfection)** | *S_R_* | $\frac{dS_{R}}{dt}=\omega R-\lambda S_{R}$ | $\frac{dS_{R}}{dt}=\omega R-\lambda S_{R}-\kappa\nu S_{R}+\omega_{V}V_{2}$ |
| **Infected without symptoms** | *I_Q_* | $\frac{dI_{Q}}{dt}=\lambda S_{R}-\delta I_{Q}$ | $\frac{dI_{Q}}{dt}=\lambda S_{R}-\delta I_{Q}$ |
| **Vaccinated (never infected)** | *V_1_* |  | $\frac{dV_{1}}{dt}=\kappa\nu S-\omega_{V}V_{1}$ |
| **Vaccinated (previously infected)** | *V_2_* |  | $\frac{dV_{1}}{dt}=\kappa\nu(S_{R}+R)-\omega_{V}V_{2}$ |

Where *N* is the total population size (i.e., the sum of the model compartments). The transmission is

$$\boldsymbol{\lambda}_{\boldsymbol{a}}\boldsymbol{(t)=}\frac{\boldsymbol{\beta}_{\boldsymbol{a}}}{\boldsymbol{N}}\sum_{\text{a}\text{∈}\text{A}} \boldsymbol{(}\boldsymbol{I}_{\boldsymbol{M}}^{\left( \boldsymbol{a} \right)}\boldsymbol{(t)+}\boldsymbol{I}_{\boldsymbol{Q}}^{\left( \boldsymbol{a} \right)}\boldsymbol{(t)+r}\boldsymbol{C}^{\left( \boldsymbol{a} \right)}\boldsymbol{(t))}$$

which is the product of the effective transmission rate, and the infections present at a time t in the model. The modification of an additional dimension of strata means that

$$\boldsymbol{\lambda}_{\boldsymbol{a,w}}\boldsymbol{(t)=}\frac{\boldsymbol{\beta}_{\boldsymbol{a,w}}}{\boldsymbol{N}}\sum_{\begin{aligned} \text{w }\text{∈}\text{ W, } \\ \text{a}\text{∈}\text{ A} \end{aligned}} \boldsymbol{(}\boldsymbol{I}_{\boldsymbol{M}}^{\left( \boldsymbol{a,w} \right)}\boldsymbol{(t)+}\boldsymbol{I}_{\boldsymbol{Q}}^{\left( \boldsymbol{a,w} \right)}\boldsymbol{(t)+r}\boldsymbol{C}^{\left( \boldsymbol{a,w} \right)}\boldsymbol{(t))}$$

The model is *dynamic* because as infections change, the transmission rate ranges. The effective transmission rate is constant, and it is related to the primary infectious number (R_0_) by the function:

$$R_{0}=\frac{\beta}{\mu+\delta}\times\left( 1+\frac{\delta\theta r}{\mu} \right)$$

So, by consequence:

$$\beta=\frac{R_{0}\times\left( \mu+\delta\right)}{\left( 1+\frac{\delta\theta r}{\mu} \right)}$$

A full derivation of R_0_ has been published before [1, 6].

The reason that the parameter is more often expressed as R_0_ is that it is more easily interpretable than *β.* Both should range from [0, ∞). At *R_0_*< 1, a pathogen would infect a few people but would disappear without an intervention, whereas when *R_0_*> 1, the pathogen is likely to cause an epidemic and is likely to become endemic. In past publications, *R_0_* has ranged from 1.93–4.83 for typhoid. See Supplementary File 1, under the tab, named “TransPar (country wealth quintile)”.

The loops going back to the compartment (dashed) are those vaccines given to people that would not change their status but would increase the number of vaccines dispensed. The purple arrows indicate waning immunity. Note that vaccination is only applied to the age strata of interest: routine vaccination at 9 months of age from the year of introduction; campaigns at 9 months to 14 years of age in the year of introduction; and periodic campaigns in the age groups of 9 months to 2 years of age and also 2 years to 14 years of age. Equity is represented by another stratum, pictured on the right; while all wealth strata contribute to the force of infection in a manner proportional to the incidence in that stratum, wealth strata are affected by the force of infection in a manner proportional to the prevalence of risk factors of typhoid.

Input disparities by wealth for sanitation and vaccination coverage

**

Figure A‑4. Disparities in coverage of improved sanitation.

The star marks the population average. Countries are ordered by GDP per capita of 2021, except for four countries (VEN, YEM, PRK and ERI) which had no data for 2021 and for which the most recent available data was used instead. The trend of sanitation is noisy across GDP per capita, potentially contributing to the weakness of the trend of ICERs across GDP per capita for MAPs (Figure 5). The values are also presented for each country in Supplementary File 1, under the tab, named “Sanitation (country WQ)”.

Figure A‑5. Disparities in coverage of MCV1 vaccination (our proxy for TCV vaccination if administered at 9 months of age) and coverage of improved sanitation.

The star marks the population average. Countries are ordered by GDP per capita of 2021, except for four countries (VEN, YEM, PRK and ERI) which had no data for 2021 and for which the most recent available data was used instead. The trend of MCV1 coverage and sanitation is noisy across GDP per capita, potentially contributing to the weakness of the trend of ICERs across GDP per capita for MAPs (Figure 5). The values are also presented for each country and each wealth quintile in Supplementary File 1, under the tab, named “Vax cov (country WQ)”.

Fitting the model with household wealth quintiles (methods)

The model, as previously published, does not include stratification of risk or vaccination coverage by wealth quintile. Here we explain how it was modified to include wealth quintiles.

While previous global analyses [7, 8] used the burden of disease from overall estimates [9, 10], they did not consider the differential incidence of disease from the differential exposure to risk factors of typhoid that are correlated with wealth (unimproved or unsafe water and sanitation). Here, we chose to use unimproved sanitation as a proxy of differential typhoid incidence by risk factor, as calculated in Figure A-6.

Abbreviation: Q, quintile.

Figure A‑6. Illustration of how we calculated the incidence of typhoid by wealth quintiles.

This assumed that social contact among wealth quintiles is represented by a simple mixture model, as contact data is not available; all wealth quintiles contribute to the force of infection in a manner proportional to the incidence of infection in the respective quintile; wealth quintiles are infected at a rate proportional to their exposure to risk factors (unimproved or shared sanitation).

Reestimation of incidence

We have incidence rate ratios for typhoid based on the presence of risk factors (lack of safe water, lack of improved sanitation) but we need odds ratios (OR) for each wealth quintile [11]. Like in other publications [12], we will determine the OR of disease for each wealth quintile as a product of the OR of the risk factor and on the prevalence of those risk factors in each quintile:

*For simplicity, suppose there is a population of 100,000 people and there are two wealth quintiles—rich or poor—partitioning the whole population into two equal halves. Wealth quintile 1 has an exposure of 40% to unimproved water sources, and WQ 2 has an exposure of 10% to unimproved water sources. Suppose the incidence risk ratio of typhoid is 2 for individuals exposed to unimproved water sources compared to the unexposed, and the incidence of typhoid has been calculated as 100 per 100,000 for the entire population. Let x be the incidence of typhoid among people with no exposure to unimproved water sources.*

*Cases among WQ 1 + Cases among WQ 2 = 100*

*Cases among WQ Y =*

*[Proportion “Have-nots” in WQ Y*Odds ratio*Incidence “haves” (unknown x) +*

*Proportion “Haves” in WQ Y*Incidence “haves” (unknown x)]*

**Proportion of whole population that is in WQ Y*

*Therefore:*

*(0.4*2*x + 0.6*x)*50,000 + (0.1*2*x + 0.9*x) *50,000 = 100*

*x is 80 per 100,000*

*The burden in WQ 1 is 56 cases among 50,000 people, and the burden in WQ 2 is 44 cases among 50,000 people. So, incidence in WQ 1 is 112 cases per 100,000 people per year, and the incidence in WQ 2 is 88 cases per 100,000 people per year.*

Reestimated incidence and effective transmission rate

The calculation in Figure A-6 gives us incidence, but not the effective transmission rate or the percent of cases that are symptomatic, which is what we need as an input for the model and to simulate vaccine impact.

In order to re-parameterize the model with as few parameters as possible, we decomposed R_0_ (which turns into the effective transmission parameter *β*) into two components.

As mentioned before,

$$R_{0}=\frac{\beta}{\mu+\delta}\times\left( 1+\frac{\delta\theta r}{\mu} \right)$$

So, by consequence:

$$\beta=\frac{R_{0}\times\left( \mu+\delta\right)}{\left( 1+\frac{\delta\theta r}{\mu} \right)}$$

And this is the new formulation of *R*_0_ (which is a linear relationship to *β)*:

$$R_{0}^{\text{new}}={exp(R}_{0}^{\text{full sanitation}}+R_{0}^{\text{multiplier}}\times\text{share population without improved sanitation)}$$

$R_{0}^{\text{full sanitation}}$ is the $R_{0}$when everyone has access to safe waste management. If $R_{0}^{\text{full sanitation}}$ < 1, that would mean that when the share of the population without improved sanitation is 0, then typhoid would eventually disappear. $R_{0}^{\text{multiplier}}$ is the term that raises the transmission in a wealth quantile according to the share of the population that does not own improved sanitation. In order to fit *R*_0_ and ensure that the values do not reach under 0, we used an exponential transformation.

Inputs for refitting:

- Incidence [9, 10].

Incidence by wealth quintile for each country, calculated as shown in Figure A-6Abbreviation: Q, quintile.

- Figure A‑6.
  - Share of the population in each wealth quintile that does not have improved sanitation, as illustrated in Figure A‑4Figure A-4.
  - Odds ratio of typhoid infection between people who have and who do not have safe sanitation: 1.56 (1.25–1.95) [11].
- Average age of infection (from Antillon et al., 2017, and IHME, [9, 10]).

In the previous publications, the average age of infection is what determines the effective transmission rate, and the incidence that is expected is what determines the percent of symptomatic cases. Because we do not expect that symptomatic cases are different by wealth quintile, we used only one parameter for symptomatic cases for all wealth quintiles, and we attribute the differential incidence to differential effective transmission [7].

In order to determine the correct effective transmission rate and the percent of cases that are symptomatic, we use a Nelder-Mead algorithm that maximizes the likelihood function shown below. The algorithm yields optimal parameters as well as a Hessian matrix, from which a covariate matrix can be calculated and standard errors of the parameters can be estimated. The overall (marginal) likelihood function is composed of two parts:

The standard errors are given by the diagonal of the inverse of the Hessian matrix that comes as part of the output with the parameters.

L(cases, parameter) = L(cases|parameters) × L(parameters)

Marginal likelihood = Conditional likelihood × Parameter likelihood

The likelihood is the product of the conditional likelihood of the cases, given a set of parameters, times the likelihood of the parameters (“priors” in Bayesian statistics). For our purposes, because we were not interested in a strictly Bayesian analysis, the purpose of this last part was only to keep the parameters within a range of values that would be sensible for the model (i.e., make sure positive parameters stay positive).

Notes Table A-1. Breakdown of the likelihood function and the terms that constitute the likelihood.

| **Operator** | **Term in the likelihood function** | **Portion of the likelihood function** | **Comments** |
| --- | --- | --- | --- |
|  | $\text{Norm}\left( \mu_{\text{burden}}, \sigma_{\text{burden}} \right)$ | Conditional likelihood | To make sure the burden overall remains the same as before. |
| $\times$ | $\text{Norm}\left( \mu_{\text{av age of inf}}, \sigma_{\text{av age of inf}} \right)$ |  | To make sure the average age of infection remains the same as before. |
| $\times$ | $\text{Dirichlet}\left( A_{WQ} \right)$ |  | Dirichlet distributions are the priors for a multinomial distribution (the generalization of the beta distribution for binomial distributions). See below the table for an explanation of how these were parameterized. |
| $\times$ | logistic(0, 1)  We wanted to limit the range of this parameter to 0.33—5 on the natural scale (after transforming by exponentiating), so we take the output of the logistic distribution (x, which ranges from 0 to 1) and multiply exp(logis(x) × 2.609 - 1). Subtracting 1 gives us a range that includes values under 1. | Likelihood of the parameter value | $R_{0}^{\text{full sanitation}}$ |
| $\times$ | logistic(0, 1)  We wanted to limit the range of this parameter to 1—10 on the natural scale (after exponentiating), so we take the output of the logistic distribution (x) and multiply exp(have-nots × logis(x) × 2.303). |  | $R_{0}^{\text{multiplier}}$ |
| $\times$ | MultNorm(< m1, m2 >, covariate matrix)  m1 and m2 have a range of 0—1, so in order to use a multivariate normal distribution, they were first transformed using a logistic function, which results in parameters on the (-∞,∞) scale, which works well with a multivariate normal distribution. |  | *m_1_* and *m_2_* – multipliers for the transmission for children 0–2 and 2–4 years old. See [6] for further explanation. |
| $\times$ | logistic(location_r_, scale_r_) |  | *r–-* the contribution to infection of secondary infections and chronic carriers. |
| $\times$ | logistic(location_rep_, scale_rep_) |  | *rep –* the share of the first-time infections that are symptomatic. |

Abbreviations: m, multiplier for the transmission of children; r, contribution to infection of secondary infections and chronic carriers; rep, share of the first-time infections that are symptomatic.

Dirichlet alphas

To derive the Dirichlet distribution of the cases, we drew 10,000 iterations of a log-normal distribution that characterized the odds ratios that a person with unmanaged sanitation would get typhoid compared to a person with managed sanitation. We calculated the incidence per wealth quintile according to the distribution of managed sanitation depicted in Figure A-6, and then calculated the proportion of all the cases in each iteration that were in each wealth quintile:

$$\text{Proportion of cases in WQ} y,\text{ iteration} i= \frac{\text{Cases in WQ }y,\text{ iteration} i}{\sum_{z} \text{Cases in WQ} z,\text{ iteration} i}$$

Using the Dirichlet method of moments, the alpha for each wealth quintile (WQ) is

$$\alpha\left( \text{WQ} y \right)=\left( \frac{E \left( \text{chosen WQ across all iterations} \right)\left( 1-E \left( \text{chosen WQ across all iterations} \right) \right)}{\text{Var} \left( c\text{hosen WQ across all iterations} \right)}-1 \right)\times\frac{E\left( \text{Cases WQ }y a\text{cross all iterations} \right)}{\text{Sum cases across all iterations}}$$

Fitting the model with wealth quintiles (results)

The results of the fitting, listed for each country, are found in Supplementary File 1 under the tab, named “TransPar (country WQ)”.

In Figure A-7, we show the incidence for the original model (without wealth quintiles) and the incidence with the re-parameterized model (with wealth quintiles). In Figure A-8, we show the target incidence for each wealth quintile, after taking into account exposure to improved sanitation, and the incidence given by the re-parameterized model for each wealth quintile.

In Figure A-9, we show the case burden across wealth quintiles, to show that the target distribution and the distribution given by the re-parameterized model were similar. Moreover, we show the distribution of cases at the beginning of MAP initiation to show how the burden would have already shifted due to N&S deployment across wealth quintiles.

Figure A‑7. Incidence per 100,000 population in the previous model (without wealth quintiles) and in the re-parameterized model with wealth quintiles.

Each dot represents one country. The previous model was that used for Bilcke et al. [7], and the new model integrates the differential exposure to typhoid in each wealth quintile via unsafe water management. This graph shows that the model re-parameterization did not change the inferences on the whole population-level typhoid incidence.

Figure A‑8. Incidence per 100,000 population in the previous model (without wealth quintiles) and in the re-parameterized model with wealth quintiles.

Each dot is equal to one country. The previous model was that used for Bilcke et al. [7], and the new model integrates the differential exposure to typhoid in each wealth quintile via unsafe water management.

Abbreviations: I, distribution of cases among wealth quintiles yielded by the model at the expected time of microarray patch initiation; M, distribution of cases among wealth quintiles yielded by the recalculated parameters of the typhoid dynamic transmission model; T, target distribution of cases among wealth quintiles according to calculations about incidence rate ratios of typhoid between people with and without safely managed wase.

Note: The vertical lines show the expected distribution under an assumption of perfect equity.

Figure A‑9. Case burden distribution among wealth quintiles. Estimates for (T) are calculated by incidence rate ratios and the prevalence of unimproved sanitation in each wealth quintile. Estimates for (M) come from the recalculated model of typhoid transmission that includes wealth quintiles. Estimates for (I) are calculated by the model at the time of MAP initiation.

Simulating MAP impact after accounting for N&S impact

*
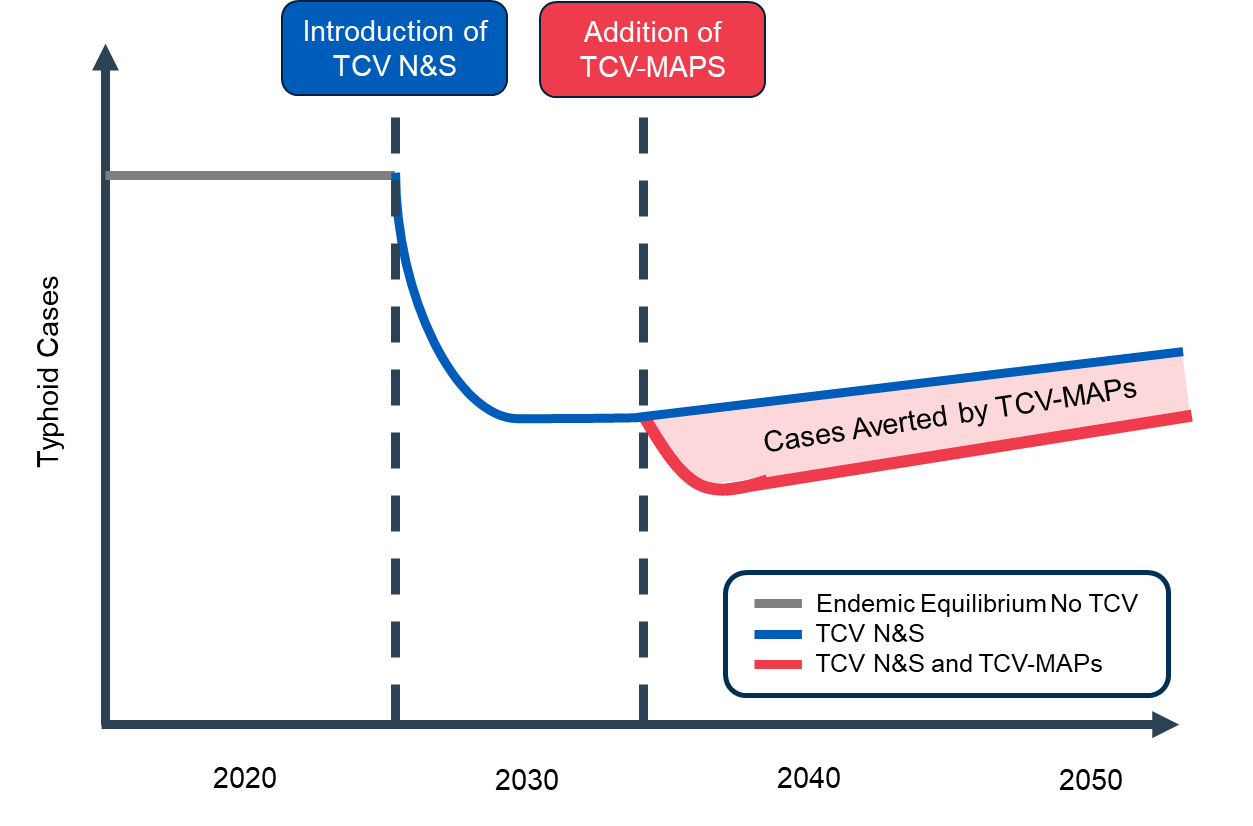
*

Abbreviations: TCV, typhoid conjugate vaccine; TCV-MAP, typhoid conjugate vaccine with microarray patch; TCV-N&S, typhoid conjugate vaccine with needle and syringe.

Figure A‑10. Illustration of the projected impact of the introduction of TCV-MAPs on the total number of cases.

The black line shows the typhoid cases at equilibrium until TCV is introduced in the decade of 2020. The blue line shows the decrease in cases once TCV -N&S is introduced. The red line shows the additional decrease that could be possible with expanded reach through MAPs.

Since the impact of TCV-MAPs depends on the extant immunity in the population, which in turn depends on lifelong exposure of the population to typhoid and accumulated immunity through previous deployment of TCV-N&S, our model projects the impact of TCV-N&S deployment before projecting the impact of TCV-MAPs, as shown in Figure A-10. Additionally, we calculate how much the TCV-N&S immunity has waned before TCV-MAPs are introduced.

**Appendix A-3:** Disease parameters and references

Table A‑3. Disease parameters, DALY inputs, and costs with references.

| **Parameter** | **Value** | **Source and rationale** |
| --- | --- | --- |
| **Demographics** | | |
| Crude birth rate. | Varies by country. | [13] See Supplementary File 1. |
| Crude death rate. | Varies by country. | [13] See Supplementary File 1. |
| Life expectancy. | Varies by country. | [13] See Supplementary File 1. |
| **Typhoid epidemiology** | | |
| Duration of infectiousness. | 4 weeks, exponential | [14] |
| Fraction infected who become chronic carriers. | Fixed, but varies by age.  < 25 years: 0.003.  >= 25 years: 0.021. | [15] |
| Duration of immunity. | 104 weeks, exponential. | [14] |
| Relative transmission rate for children 0–2 years. | Varies by country. | Result of refit exercise. Input to the dynamic model. See Supplementary File 1. |
| Relative transmission rate for children 2–5 years. | Varies by country. | Result of refit exercise. Input to the dynamic model. See Supplementary File 1. |
| Relative infectiousness of chronic carriers. | Varies by country. | The prior is estimated to reproduce the indirect protection observed in a cluster-randomized trial of Vi-polysaccharide vaccine [6].  Result of refit exercise. Input to the dynamic model. See Supplementary File 1. |
| Mean age at infection. | Varies by country. | Output from the dynamic model. See Supplementary File 1. |
| Incidence. | Varies by country and wealth quintile. | Output from the dynamic model. See Supplementary File 1. |
| *R*_0_ (reproductive number). | Varies by country and wealth quintile. | Result of refit exercise. Input to the dynamic model. See Supplementary File 1. |
| Percent symptomatic. | Varies by country. | Result of refit exercise. Input to the dynamic model. See Supplementary File 1. |
| AMR prevalence. | Varies by country and when not available, by UN subregion. | Used the sum for MDR and XDR from [16]. See Supplementary File 1. |
| **Treatment related** | | |
| Probability of infected patients seeking health care, no AMR. | 0.57 | Based on [6]; based on estimate of relative incidence for passive versus active surveillance. |
| Probability of infected patients seeking health care, no AMR. | 0.72 | Based on [7], we assumed that the odds of seeking care double if patient has an antimicrobial-resistant strain. |
| Proportion of care-seeking patients become inpatients, no AMR. | 0.14 | Based on [17-19]. |
| Proportion of care-seeking patients become inpatients, with AMR. | 0.25 | Based on [7], we assumed that the odds of hospitalization double if the patient has an antimicrobial-resistant strain. |
| Probability that non-care-seeking patient is severe, no AMR. | 0.10 | Half the probability of hospitalization.  Source: assumption. |
| Probability that non-care-seeking patient is severe, with AMR. | 0.18 | Half the probability of hospitalization.  Based on [7], we assumed that the odds of hospitalization double if the patient has an antimicrobial-resistant strain. |
| Number of visits to a medical doctor by outpatients. | 1 | Fixed, assuming that the costs of a clinical visit are low and therefore unlikely to significantly affect the results. |
| Relative duration of illness for patients not seeking medical care (versus inpatients and outpatients). | 0.5 | Assumption. |
| Probability of intestinal perforation:  Africa  Asia  Elsewhere | 0.076  0.007  0.007 | Based on [20]. |
| CFR—no intestinal perforation:  Africa  Asia  Elsewhere | 0.062  0.010  0.010 | Based on [20].  Assumption for elsewhere, as no information is available regarding mortality outside of Asia or Africa. |
| CFR—with intestinal perforation:  Africa  Asia  Elsewhere | 0.197  0.048  0.01 | Based on [20].  Assumption for elsewhere, as no information is available on intestinal perforation outside of Asia or Africa. |
| CFR—no health care seeking. | 1.5 times CFR for patients without intestinal perforation. | Assumption. Results in approximately a third of deaths being outside the reach of the health care system. See notes below this table. |
| **Costs** | | |
| Outpatient drug costs per course:  Asia  Mideast  Africa  Eurasia  Americas | 13.38  10.13  0.35  10.13  10.13 | Asia: [21-24].  Africa: [25-27].  Other regions: average of all aforementioned papers. |
| Inpatient costs:  Asia  Mideast  Africa  Eurasia  Americas | 143.81  171.48  226.81  171.48  171.48 |  |
| Outpatient costs:  Asia  Mideast  Africa  Eurasia  Americas | 47.15  42.19  32.26  42.19  42.19 |  |
| Intestinal perforation costs:  Asia  Mideast  Africa  Eurasia  Americas | 191.27  183.08  246.83  183.08  183.08 |  |
| Inpatient drug costs per course:  Asia  Mideast  Africa  Eurasia  Americas | 64.70  62.61  56.35  62.61  62.61 |  |
| **DALY inputs** | | |
| Disability weights:  moderate illness  severe illness  intestinal perforation | 0·052  0·210  0.324 | [28] |
| Duration of illness in inpatients and outpatients without intestinal perforation (days). | 16 | Based on [29-31]; SE is based on the prediction interval of random effects meta-analysis. See calculation in the supplement of [7]. |
| Duration of illness in inpatients with intestinal perforation (days) | 32 | [23] |
| Age at death from typhoid infection. | Varies by country. | Output of cases of each age group from the dynamic model times the midpoint age of the age group. |

Abbreviations: AMR, antimicrobial resistance; CFR, case fatality rate; DALY, disability-adjusted life year; MDR, multi-drug resistant; SE, standard error; UN, United Nations; XDR, extensively drug resistant.

Parameter notes

Hospitalization

This is admission to the hospital and not just care-seeking in outpatient departments of hospitals. To remain current, we have only taken into account estimates from studies since 2010. In India, hospitalization was estimated as 15% across sites [17]. Estimates from Nepal, Pakistan, and Bangladesh were much higher, ranging from 27% in Bangladesh, 28% in Nepal, and 47% in Pakistan [18]. Three other recent studies available had the hospitalization probability at 3% in Bangladesh and Nepal and 8% in Malawi [19].

Carias et al., in Uganda, uses an estimate of 32% for cases without intestinal perforation [26]. Although this estimate was not used in our calculation because it was an estimate gathered from “expert opinion”, we mention it here to show the range of possible estimates.

We used random effects estimate of 14% because the test of heterogeneity has a significant value.


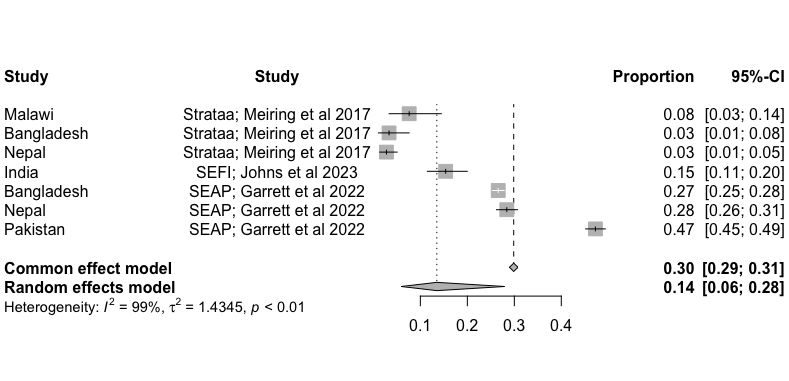


Abbreviations: SEAP, Surveillance for Enteric Fever in Asia Project; SEFI, Surveillance of Enteric Fever in India; STRATAA, Strategic Typhoid Alliance Across Africa and Asia.

Notes Figure A-1. Meta-analysis to calculate the probability of hospitalization that we use in the probability tree model.

CFR: no health care–seeking

Bilcke et al. assumed that the proportion of cases occurring out of care varies between 0% and 75%, with an average of 0.38 [7]. However, this was an assumption with no basis, as there is no good surveillance that we know of that investigates mortality outside of care.

We applied a factor of 1.5 to the typical CFR to estimate deaths outside of care among severe cases, which we already assumed are only half as likely as severity among cases that seek care. The assumption is that a case that does not receive care is more likely to become fatal than a case that does seek care. We assume that these probabilities—of seeking care and of being severe—would double in their odds (not probabilities) for cases with antimicrobial-resistant disease, since the first medicines acquired would not work, and presumably patients would then seek care. We double the odds rather than the probabilities in order to keep probabilities within the range of [0–1]. See the table of parameters for the resulting probabilities.

In conclusion, using a factor of 1.5 on the odds of death and taking into account AMR and care-seeking, 0.26 of all deaths in Malawi will be out of care, and 0.34 of all deaths in Nepal would be outside of care, the two example countries shown in Appendix B-2. For perspective, this is slightly lower than the estimate by Bilcke et al. [7], but higher than a study of enteric fever in India that found that 25.8% of deaths associated with febrile illness had not sought any care, although this study was not able to confirm the pathogen associated with these deaths [7, 32].

Vaccine efficacy (probability of protection upon vaccination and duration)

Only four studies so far measure the efficacy of TCV-N&S. We chose a vaccine efficacy of 85%, which is consistent with current studies, as shown in the table below.

Notes Table A-2 Literature estimates of vaccine efficacy for typhoid conjugate vaccines.

| **Country** | **Context** | **Design** | **Point estimate** | **Citation** |
| --- | --- | --- | --- | --- |
| Nepal | TyVAC | Randomized | 79% (95% CI, 62–89) | [33] |
| Bangladesh | TyVAC | Cluster-randomized | 85% (97.5% CI, 76–91) | [34] |
| Malawi | TyVAC | Randomized | 81% (95% CI, 64–90) | [35] |
| Pakistan | XDR outbreak | Randomized | 95% (95% CI, 93–96) | [36] |

Abbreviations: CI, confidence interval; TyVAC, Typhoid Vaccine Acceleration Consortium; XDR, extensively drug resistant.

For duration, we assumed a 10-year period for both TCV-N&S and for TCV-MAPs. Thus far, only one study exists measuring the duration of TCV protection, which was estimated to be four years [37].

**Appendix A4:** TCV historical introduction

Table A‑4. TCV introduction history. TCV-N&S adoption dates and strategy for countries currently deploying TCV-N&S.

Adapted from private communication from Ashley Latimer, PATH, 11 March 2024, and Nampota-Nkomba et al. [38]. Pakistan, Liberia, Zimbabwe, Nepal, and Malawi are Gavi-eligible countries. Samoa and Fiji are upper-middle-income countries and are therefore not Gavi eligible.

| Country | Introduction Strategy | Age |
| --- | --- | --- |
| Pakistan | Nationwide campaign, followed by routine immunization. | Campaign: 9 months–15 years  Routine: 9 months |
| Liberia | Nationwide campaign, followed by routine immunization. | Campaign: 9 months–15 years  Routine: 9 months |
| Zimbabwe | Nationwide campaign, followed by routine immunization. | Campaign: 9 months–15 years  Routine: 9 months |
| Nepal | Nationwide campaign, followed by routine immunization. | Campaign: 9 months–15 years  Routine: 15 months |
| Malawi | Nationwide campaign, followed by routine immunization. | Campaign: 9 months–15 years  Routine: 15 months |
| Samoa [39] | Nationwide campaign, followed by a routine immunization. | Campaign: 12 months–45 years, including older ages for high-risk groups.  Routine: 12 months |
| Fiji [40] | Campaign in the Northern Division, aimed at elimination. | Campaign: 9 months–65 years |

**Appendix A-5:** Relative proportion of TCV recipients that are military personnel (UC 5) and travelers (UC 6)

In a population of 100,000 people in Nigeria, the proportion of recipients would be as follows:

- Children, annually, who turn 9 months old (for recipients in UC 1–UC 3)*: 3,160.
- Coverage, 60%: 1,896.
- Adults, age 16–45, who belong to the military (UC 5), which is 114% of the whole population [41]: 114.
  - - Vaccinated in UC 5, 10%: 11.
- Adults, age 15+ (UC 6): 20,019.
  - Eligible, who travel and would be vaccinated for typhoid, 1%: 200.
    - Vaccinated in UC 6, 20%: 40.

Therefore, in Nigeria, the percentage of TCV recipients who are adults is (11 + 40)/(1,896 + 11 + 40) = 2.6%.

*UC 4 is a one-time campaign in the first year of the analytic horizon among children 9 months to 15 years of age if TCV has not been introduced, but in our base-case analysis, we assume that TCV has been introduced in all countries, so we calculate this population to be 0.

Sensitivity analysis

Nigeria’s population has among the smallest shares of military personnel (ranking 150 out of 170 countries). There are countries with very large shares of the population that belong to the military (e.g., North Korea has the largest share at 5.8%, Eritrea at 4.5%, and Laos at 2.3%). These three countries have a “low-income country” population distribution in the model. (For technical reasons, we did not do different distributions for different countries; see Bilcke et al. [7].)

Therefore, there would still be 3,160 children, annually, who turn 9 months old, but the coverage in North Korea, Eritrea, and Laos is 99.7%, 84.2%, and 66%, respectively, or 3,151, 2,661, and 2,086 children vaccinated. We can assume that 40 travelers per 100,000 would still be vaccinated, but there would be military populations of 5,800 in North Korea, 4,500 in Eritrea, and 2,300 in Laos. Only 10% of these military personnel would be vaccinated, which would yield 580, 450, and 230 vaccinated adults, in addition to the 40 travelers. This would yield a percentage of TCV recipients who are adults at 16%, 16%, and 11%, respectively.

**Appendix A-6:** TCV product distribution by country per use case

Figure A‑11. Coverage of population by use case before MAPs introduction by country.

The values are also presented for each country in Supplementary File 1, under the tab, named “Vax cov (country WQ)” combined with the information from the tab, named “Vax cov use cases (country)”.

**Appendix A-7:** MCV coverage per country per use case, apportioned to the different wealth quintiles

Table A‑5. Example of Nigeria, how we combine information on use cases and vaccination by wealth quintile to insert it into a model with both mechanisms.

We assumed that the population of each quintile reached via each use case was proportional to both the coverage of vaccination in each wealth quintile and the proportion of the total population reached via each use case.

| **Use case** | **National mean** | **WQ 1** | **WQ 2** | **WQ 3** | **WQ 4** | **WQ 5** |
| --- | --- | --- | --- | --- | --- | --- |
| **MCV1 coverage (DHS)** | **60%** | **44%** | **50%** | **61%** | **70%** | **87%** |
| **UC 1: fixed post**  **85%** of < 2 years old reached via fixed post | 85% x 60% = 51% | 85% x 44% = 37.4% | 85% x 50% = 42.5% | 85% x 61% = 52.85% | 85% x 70% = 69.5% | 85% x 87% = 73.95% |
| **UC 2: outreach**  **10%** of < 2 years old reached via outreach | 10% x 60% = 6% | 10% x 44% = 4.4% | 10% x 50% = 5% | 10% x 61% = 6.10% | 10% x 70% = 7% | 10% x 87% = 8.70% |
| **UC 3: mobile**  **5%** of < 2 years old reached via mobile | 5% x 60% = 3% | 5% x 44%= 2.2% | 5% x 50% = 2.5% | 5% x 61% = 3.05% | 5% x 70% = 3.5% | 5% x 87% = 4.35% |
| **UC 4: 2- to 15-year-olds** |  | N/A in Nigeria; only for countries with no N&S before 2032. | | | | |
| **UC 5: military**  **(< 1%** of pop 15–45) |  | 10% | | | | |
| **UC 6: travelers**  **(< 1%** of pop 15+) |  | 20% | | | | |

Abbreviations: DHS, Demographic and Health Surveys; MCV, measles-containing vaccine; N/A, not applicable; N&S, needle and syringe; pop, population; UC, use case; WQ, wealth quintile.

Here we provide the example of Nigeria, to show how we combine information on use cases with the different market penetration scenarios to calculate how TCV-N&S and MAP supplies are distributed. The calculations and totals are in Notes Table 3.

As explained in the main text, the potential deployment scenarios are:

- Comparator (TCV-N&S): TCV-N&S when no MAPs is deployed, as seen in Table A-5.
- TCV-MAPs mix scenario 1: 80% replacement of TCV-N&S with TCV-MAPs in all use cases
- TCV-MAPs mix scenario 2: 100% replacement of TCV-N&S with TCV-MAPs in use cases 2 and 3, but 100% of TCV-N&S vaccinations **remain** in use case 1.
  - **All new coverage** (20% of the unreached population) is given MAPs and reached via mobile and outreach activities (use cases 2 and 3).
  - Note: To calculate how much of the new coverage is distributed via use case 2 and use case 3, we have the percent of new coverage that will go to use case 2 and use case 3, as a proportion of the coverage in both case 2 and 3 together:

$$\frac{\text{Coverage in use case 2}}{\text{Coverage in use case 2 + Coverage in use case 3}}$$

$$\frac{\text{Coverage in use case 3}}{\text{Coverage in use case 2 + Coverage in use case 3}}$$

- TCV-MAPs mix scenario 3: 100% replacement of TCV-N&S with TCV-MAPs
  - All new coverage receives MAPs.

In summary, this means that under the comparator, the overall coverage is 60% (Table A-5 and Notes Table 3) but that under any of the TCV-MAPs mix scenarios coverage rises to 68% (vaccinated population + 20% of currently unvaccinated population). What sets the different mix scenarios apart is the way in which TCVs are deployed (by different use cases which has delivery cost implications) and the penetration of TCV-MAPs in the population <3 years of age. Under mix scenario 1, 82% of all vaccinated individuals will be vaccinated by TCV-MAPs, whereas the penetration is 25% and 100% in mix scenarios 2 and 3 respectively. For more details, see Notes Table A-3.

Notes Table A-3. Distribution of TCV-N&S and TCV-MAPs in old and new coverage by use case and by TCV-MAPs mix scenario.

|  | **Previous coverage that remains with TCV-N&S: 20%** | **Previous coverage that switches to TCV-MAPs: 80%** | **New coverage with TCV-MAPs: 20% of uncovered** |
| --- | --- | --- | --- |
| ***Calculations*** | | | |
| ***Comparator (TCV-N&S only)*** | | | |
| *Use case 1*  *(85% of coverage)* | 85%  x 60%  x 100%  = 51% | 0% | 0% |
| *Use case 2*  *(10% of coverage)* | 10%  x 60%  x 100%  =6% | 0% | 0% |
| *Use case 3*  *(5% of coverage)* | 5%  x 60%  x 100%  =3% | 0% | 0% |
| ***TCV-MAPs mix scenario 1*** | | | |
| *Use case 1*  *(85% of coverage)* | 85%  x 60%  x (100%-80%)  = 10.20 | 85%  x 60%  x 80%  = 40.80 | 85%  x (100%-60%) {unvax}  x 20% {cov of unvax}  =6.60 |
| *Use case 2*  *(10% of coverage)* | 10%  x 60%  x (100%-80%)  =1.20% | 10%  x 60%  x 80%  =4.80% | 10%  x (100%-60%) {unvax}  x 20% {cov of unvax}  =0.80% |
| *Use case 3*  *(5% of coverage)* | 5%  x 60%  x (100%-80%)  =0.60% | 5%  x 60%  x 80%  =2.40% | 5%  x (100%-60%) {unvax}  x 20% {cov of unvax}  =0.40% |
| ***TCV-MAPs mix scenario 2*** | | | |
| *Use case 1*  *(85% of coverage)* | 85%  x 60%  x 100%  = 51% | 0% | 0% |
| *Use case 2*  *(10% of coverage)* | 0% | 10%  x 60%  x 100%  = 6% | 10%/(10% + 5%)  x (100%-60%) {unvax}  x 20% {cov of unvax}  = 5.33% |
| *Use case 3*  *(5% of coverage)* | 0% | 5%  x 60%  x 100%  = 3% | 10%/(10% + 5%)  x (100%-60%) {unvax}  x 20% {cov of unvax}  = 2.67% |
| **TCV-MAPs mix scenario 3** | | | |
| *Use case 1*  *(85% of coverage)* | 0% | 85%  x 60%  x 100%  = 51% | 85%  x (100%-60%) {unvax}  x 20% {cov of unvax}  =6.60 |
| *Use case 2*  *(10% of coverage)* | 0% | 10%  x 60%  x 100%  = 6% | 10%  x (100%-60%) {unvax}  x 20% {cov of unvax}  =0.80% |
| *Use case 3*  *(5% of coverage)* | 0% | 5%  x 60%  x 100%  = 3% | 5%  x (100%-60%) {unvax}  x 20% {cov of unvax}  =0.40% |
| ***Subtotals by product and coverage (old or new)*** | | | |
| *Comparator (TCV-N&S)* | 60% | 0% | 0% |
| *TCV-MAPs mix 1* | 12% | 48% | 8% |
| *TCV-MAPs mix 2* | 51% | 9% | 8% |
| *TCV-MAPs mix 3* | 0% | 60% | 8% |
| ***Subtotals by product*** | | | |
| *Comparator (TCV-N&S)* | 60% | 0% | |
| *TCV-MAPs mix 1* | 12% | 56% | |
| *TCV-MAPs mix 2* | 51% | 17% | |
| *TCV-MAPs mix 3* | 0% | 68% | |
| **Total coverage** | | | |
| *Comparator (TCV-N&S)* | 60% | | |
| *TCV-MAPs mix 1* | 68% | | |
| *TCV-MAPs mix 2* | 68% | | |
| *TCV-MAPs mix 3* | 68% | | |
| **Effective MAPs penetration in population < 2** | | | |
| *Comparator (TCV-N&S)* | 0% | | |
| *TCV-MAPs mix 1* | 82% | | |
| *TCV-MAPs mix 2* | 25% | | |
| *TCV-MAPs mix 3* | 100% | | |

Abbreviations: MAP, microarray patch; TCV-MAP, typhoid conjugate vaccine with microarray patch; TCV-N&S, typhoid conjugate vaccine with needle and syringe; cov, coverage; unvax =unvaccinated.

**Appendix A-8**: Sensitivity analysis: TCV coverage by wealth quintile (MCV2 coverage as a proxy)

Abbreviations: DHS, Demographic and Health Surveys; MCV, measles-containing vaccine.

Figure A‑12. MCV1 and MCV2 coverage levels by country and wealth quintile.

MCV1 coverage is used as the proxy for TCV coverage that would be achieved if administered at 9 months of age, and MCV2 coverage is used as a proxy of the TCV coverage that would be achieved if administered at 15 months of age.

**Appendix A-9:** Characteristics of the countries in the subnational analysis

Table A‑6. Characteristics of countries in the subnational analysis.

| **Characteristics** | **Burkina Faso** | **India** | | **Kenya** | **Malawi** | | **Nepal** |
| --- | --- | --- | --- | --- | --- | --- | --- |
| **General** | | | | | | | |
| Archetype | 2 | 5 | | 2 | 3 | | 5 |
| Income classification | Low income | Lower-middle income | | Lower- middle income | Low income | | Lower- middle income |
| GDP per capita | 833 | 2,389 | | 2,099 | 645 | | 1,337 |
| Number of districts/counties^a^ | 13 | 707 | | 47 | 32 | | 77 |
| Total population | 21,134,586 | 1,371,360,350 | | 47,564,296 | 19,351,892 | | 29,192,480 |
| TCV-MAPs adoption year | 2034 | 2035 | | 2034 | 2034 | | 2034 |
| **Demographic** | | | | | | | |
| Median crude birth rate  (births per 1,000 population) | 36.085 | 16.572 | | 27.998 | 33.233 | | 20.64 |
| Median crude death rate  (deaths per 1,000 population) | 8.813 | 7.35 | | 7.456 | 6.706 | | 7.209 |
| Life expectancy at birth (2019) | 61.6 | 69.7 | | 66.7 | 64.3 | | 70.8 |
| **Typhoid epidemiology** | | | | | | | |
| Mean age at infection | 12.37 | 13.70 | | 13.31 | 12.92 | | 13.21 |
| Mean reproduction rate (R_0_) [CI] | 2.50  [2.39–2.61] | 3.53  [3.43–3.64] | | 2.32  [2.15–2.48] | 2.40  [2.30–2.50] | | 3.70  [3.53–3.87] |
| Mean symptomatic probability [CI] | 0.19  [0.17–0.22] | 0.16  [0.05–0.27] | | 0.15  [0.08–0.21] | 0.08  [0.04–0.12] | | 0.15  [0.07–0.24] |
| Mean incidence [CI] (2015) | 495  [422–567] | 298  [92–509] | | 353  [201–510] | 196  [96–293] | | 289  [124–453] |
| Prevalence of AMR | 0.01 | 0.03 | | 0.78 | 0.93 | | 0.02 |
| Mortality rate (treated, sensitive, hospitalized) | 0.062 | 0.01 | | 0.062 | 0.062 | | 0.01 |
| Mortality rate (treated, resistant, hospitalized) | 0.124 | 0.02 | | 0.124 | 0.02 | | 0.124 |
| Mortality rate (treated intestinal perforation) | 0.197 | 0.046 | | 0.197 | 0.197 | | 0.046 |
| Probability of intestinal perforation | 0.076 | 0.007 | | 0.076 | 0.076 | | 0.007 |
| CFR | 0.0086 | 0.00137 | | 0.0233 | 0.0261 | | 0.00134 |
| **Prevalence risk factors** | | | | | | | |
| MCV1 coverage by district (range) | 0.85  (0.76–0.94) | 0.88  (0.53–1.00) | | 0.88  (0.41–0.99) | 0.88  (0.78–1.00) | | 0.88  (0.57–1.00) |
| Improved sanitation coverage (not shared) by district (range) | 0.38  (0.22–0.72) | 0.70  (0.25–1.00) | | 0.41  (0.09–0.77) | 0.46  (0.28–0.77) | | 0.71  (0.34–0.91) |
| MCV1 coverage by wealth quintile (whole country) | 0.85  (0.83–0.86) | 0.88  (0.84–0.91) | | 0.88  (0.79–0.92) | 0.88  (0.85–0.92) | | 0.88  (0.83–0.93) |
| Improved sanitation coverage (not shared) by wealth quintile (whole country) | 0.38  (0.13–0.63) | 0.70  (0.38–0.96) | | 0.41  (0.11–0.71) | 0.46  (0.23–0.59) | | 0.71  (0.65–0.86) |
| **Costs ($US)** | | | | | | | |
| Mean vaccine cost—routine (2016) (range) | 1.76  (0.36–4.23) | 1.53  (0.31–3.68) | | 1.76  (0.36–4.23) | 1.76  (0.36–4.23) | | 1.53  (0.31–3.68) |
| Mean vaccine cost—campaign (2016) (range) | 0.56  (0.38–0.77) | 0.41  (0.23–0.62) | | 0.41  (0.40–0.62) | 0.41  (0.23–0.62) | | 0.41  (0.23–0.62) |
| Outpatient costs | 16.41 | 32.26 | 20.26 | | 60.35 | 32.26 | |
| Inpatient costs | 90.04 | 226.81 | 98.99 | | 397.48 | 226.81 | |
| Intestinal perforation costs | 304.95 | 246.83 | 301.60 | | 246.83 | 246.83 | |
| Drug costs—outpatient | 13.38 | 0.35 | 8.60 | | 0.35 | 0.35 | |
| Drug costs—inpatient | 64.70 | 56.35 | 51.16 | | 56.35 | 56.35 | |
| Delivery costs TCV-N&S | 0.11–1.07 | 0.11–0.86 | | 0.20–2.20 | 0.11–1.01 | | 0.12–1.84 |
| Delivery costs TCV-MAPs | 0.24–0.62 | 0.37–0.41 | | 0.35–1.95 | 0.26–0.53 | | 0.27–2.13 |

Abbreviations: AMR, antimicrobial resistance; CFR, case fatality rate; CI, confidence interval; GDP, gross domestic product; MCV, measles-containing vaccine; TCV-MAP, typhoid conjugate vaccine with microarray patch; TCV-N&S, typhoid conjugate vaccine with needle and syringe.

^a^ For Burkina Faso, no data was available at the district/county level; instead, regional data was used.

**Appendix B**

**Supplementary Results: Global Analysis**

**Appendix B-1:** Impact analysis

Table B‑1. Total cases averted by TCV-N&S and TCV-MAP presentation conditioning on different assumptions about vaccine deployment.

The first period, roughly from TCV-N&S deployment (2023 to 2032) until MAP deployment (after 2032), only one presentation is available: TCV-N&S. The second period, after 2032, refers to the period when both TCV-N&S and TCV-MAP presentations are available. The cases averted during the first period are counted over a span of years that varies by country bounded by the years of TCV-N&S deployment and MAP deployment. The cases averted during the second period are counted for 20 years after MAP deployment. MCV1 coverage is assumed to be the TCV baseline coverage because both vaccines are administered at 9 months of age. Sensitivity analyses were run with (1) MCV1 improvement assumptions in line with Market Information for Access to Vaccines (MI4A) growth rates assumptions, which would project that approximately 40% of currently unvaccinated children will be vaccinated by 2042, and (2) MCV2 coverage assumptions, which assume that the coverage of TCV will be equal to the coverage of the second MCV-containing vaccine in immunization programs, usually delivered at 15 months of age.

|  | | | | **Cases averted** | |
| --- | --- | --- | --- | --- | --- |
| **Period** | **TCV-N&S**  **coverage** | **TCV-N&S**  **deployment strategy** | **TCV-MAPs coverage of unvaccinated** | **TCV-N&S or existing coverage** | **MAPs-driven improvements in coverage** |
| 2023–2032 (No TCV = 174.65M cases) | MCV1 (default) | Routine & Campaign (default) |  | 63.11M |  |
|  |  | Routine (sensitivity) |  | 34.26M |  |
|  | MCV1 improved (sensitivity) | Routine & Campaign (default) |  | 68.56M |  |
|  |  | Routine (sensitivity) |  | 37.01M |  |
|  | MCV2 (sensitivity) | Routine & Campaign (default) |  | 43.58M |  |
|  |  | Routine (sensitivity) |  | 24.45M |  |
| 2033–2052  (No TCV = 330.66M cases) | MCV1 (default) | Routine & Campaign (default) | 20% (default) | 48.89M | 5.18M |
|  |  |  | 10% (sensitivity) |  | 2.59M |
|  |  |  | 30% (sensitivity) |  | 7.76M |
|  |  | Routine (sensitivity) | 20% (default) | 63.96M | 5.07M |
|  |  |  | 10% (sensitivity) |  | 2.54M |
|  |  |  | 30% (sensitivity) |  | 7.59M |
|  |  | None (sensitivity) | 20% (default) |  | 102.17M |
|  |  |  | 10% (sensitivity) |  | 99.07M |
|  |  |  | 30% (sensitivity) |  | 105.29M |
|  | MCV1 improved (sensitivity) | Routine & Campaign (default) | 20% (default) | 56.43M | 3.1M |
|  |  | Routine (sensitivity) |  | 72.37M | 3.02M |
|  |  | None (sensitivity) |  |  | 111.14M |
|  | MCV2 (sensitivity) | Routine & Campaign (default) |  | 31.85M | 11.11M |
|  |  | Routine (sensitivity) |  | 42.44M | 11.01M |
|  |  | None (sensitivity) |  |  | 77.71M |

Abbreviations: M, million; MAP, microarray patch; MCV, measles-containing vaccine; N&S, needle and syringe, TCV, typhoid conjugate vaccine; TCV-MAP, typhoid conjugate vaccine with microarray patch; TCV-N&S, typhoid conjugate vaccine with needle and syringe.

**Appendix B-2:** Case outcomes

97.2% antimicrobial sensitive cases

2.8% antimicrobial resistant cases

97% of DALYs

99.8% recoveries

0.2% deaths

3% of DALYs

99.4% of costs

0.6% of costs

7% antimicrobial sensitive cases

93% antimicrobial resistant cases

99% of DALYs

97% recoveries

3% deaths

1% of DALYs

90% of costs

10% of costs

Abbreviations: DALY, disability-adjusted life year, IP, intestinal perforation.

Figure B‑1. Disease outcome model with outputs, illustrated for Nepal (top) and Malawi (bottom).

In Nepal, the contribution of antimicrobial-resistant typhoid strains is less pronounced than what we may see in other countries (2.8% of all cases). The vast majority of typhoid cases result in recovery (99.8%). Total health system costs associated with cases resulting in recovery are therefore also much higher (99.8%) as compared to costs of cases leading to death. Deaths, however, represent a large proportion of DALYs due to typhoid infection (97%). In Malawi, which contends with far higher AMR cases as well as a higher CFR, there are slightly more deaths, 3%, and the deaths account for slightly higher amounts of costs, 10%, and a slightly higher share of DALYs, 99%.

**Appendix B-3:** Sensitivity analysis: product characteristics

Abbreviation: MAP, microarray patch.

Figure B‑2. Cost-effectiveness by vaccine price, MAP profile, and comparator (unweighted).

The column marked with a black box represents the results under our default assumptions. Here, we show the unweighted results; that is, a simple percentage of the countries in each cost-effectiveness category shown in the legend. Figure 3 shows the percentage of countries weighted by population size; that is, across all countries, the population that belongs to countries in each of the cost-effectiveness categories shown in the legend.

**Appendix B-4:** Sensitivity analysis: program characteristics

Figure B‑3. Cost-effectiveness by MAP coverage, vaccine price, and MAP profile (assuming 80% switch to TCV-MAPs).

Columns indicated in black boxes represent the default assumptions. MAP coverage refers to the proportion of the currently unvaccinated individuals who would be vaccinated if MAPs were introduced. On the left, the unweighted results are shown; that is, a simple percentage of the countries in each cost-effectiveness category shown in the legend. On the right, the weighted results are shown, which represent the percentage of countries weighted by population size; that is, across all countries, the population that belongs to countries in each of the cost-effectiveness categories shown in the legend.

Figure B‑4. Cost-effectiveness by TCV-N&S deployment strategy, vaccine price, and MAP profile (assuming 80% switch to TCV-MAPs).

Columns indicated in black boxes represent the default assumptions. On the left, the unweighted results are shown; that is, a simple percentage of the countries in each cost-effectiveness category shown in the legend. On the right, the weighted results are shown, which represent the percentage of countries weighted by population size; that is, across all countries, the population that belongs to countries in each of the cost-effectiveness categories shown in the legend.

Abbreviation: MCV, measles-containing vaccine.

Figure B‑5. Cost-effectiveness by MCV coverage, vaccine price, and MAP profile (assuming 80% switch to TCV-MAPs).

Columns indicated in black boxes represent the default assumptions. On the left, the unweighted results are shown; that is, a simple percentage of the countries in each cost-effectiveness category shown in the legend. On the right, the weighted results are shown, which represent the percentage of countries weighted by population size; that is, across all countries, the population that belongs to countries in each of the cost-effectiveness categories shown in the legend.

**Appendix B-5:** Inequality

Disparity before MAPs and reduction in inequality in terms of excess cases and DALYs

Abbreviations: DALY, disability-adjusted life year; MAP, microarray patch.

Figure B‑6. Reduction in inequality mapped against the expected inequality when MAPs are to be deployed.

The left panel has a correlation coefficient of 0.26 (p = 0.003), and the right panel has a correlation coefficient of 0.22 (p = 0.009).

Disparity before MAPs as measured by reduction in cases and DALYs against ICERs

Abbreviations: DALY, disability-adjusted life year; GDP, gross domestic product; ICER, incremental cost-effectiveness ratio.

Figure B‑7. ICERs, scaled by GDP per capita, mapped against the expected reduction in inequality by MAPs introduction.

The red, dashed line shows the cost-effectiveness threshold (scaled ICER of 3). The left panel has a correlation coefficient of –0.49 (p < 0.001), and the right panel has a correlation coefficient of –0.50 (p < 0.001). Analyses shown here are making default assumptions (new coverage is 20% of unvaccinated individuals, the price of the MAP is $3.00, and market penetration of MAPs is 80% across all use cases).

**Appendix B-6:** Drivers of cost-effectiveness

**Figure B‑8.** The relationship between inputs and ICERs, scaled by GDP per capita.

The blue bars represent the coefficients from a log-linear model where each of the inputs are standardized by mean and standard deviation. In that way, the variance of the log-transformed, scaled ICER is explained by the variance in the inputs, rather than the scale or range of these predictors on the natural scale (e.g. if age of infection varies 0-20 and another variable ranges 0-1, this will not matter in the standardized regression).

**Appendix C**

**Supplementary results: Subnational analysis**

**Appendix C-1:** Maps for the subnational analysis

Abbreviations: CE, cost-effective; CS, cost-saving; HCE, highly cost-effective; VCE, very cost-effective.

Figure C‑1. Cost-effectiveness by region in Burkina Faso by MAP profile and vaccine price.

The panel marked with a black box represents the results of our default assumptions.

Abbreviations: CE, cost-effective; CS, cost-saving; HCE, highly cost-effective; VCE, very cost-effective.

Figure C‑2. Cost-effectiveness by district in India by MAP profile and vaccine price.

The panel marked with a black box represents the results of our default assumptions.

Abbreviations: CE, cost-effective; CS, cost-saving; HCE, highly cost-effective; VCE, very cost-effective.

Figure C‑3. Cost-effectiveness by district in Kenya by MAP profile and vaccine price.

The panel marked with a black box represents the results of our default assumptions.

Abbreviations: CE, cost-effective; CS, cost-saving; HCE, highly cost-effective; VCE, very cost-effective.

Figure C‑4. Cost-effectiveness by district in Malawi by MAP profile and vaccine price.

The panel marked with a black box represents the results of our default assumptions.

Abbreviations: CE, cost-effective; CS, cost-saving; HCE, highly cost-effective; VCE, very cost-effective.

Figure C‑5. Cost-effectiveness by district in Nepal by MAP profile and vaccine price.

The panel marked with a black box represents the results of our default assumptions.

**Appendix C-2**: Drivers of cost-effectiveness for subnational analysis

There is no universal factor that can be used to determine cost-effectiveness across subnational regions in the countries assessed. An assessment of each parameter (i.e., poverty, sanitation, and vaccine coverage), using scaled ordinary least squares (OLS) regression to understand the individual and joint effects on the ICERs within each country, is shown in Table C-1. Negative correlations show that ICERs decrease with increasing values of the predictor; that is, the intervention becomes more favorable. Positive correlations show that the ICER increases with increasing values of the predictor; that is, the intervention becomes less favorable. In general, we would expect negative correlations with poverty and positive correlations with higher sanitation coverage and higher MCV1 coverage.

Despite being relatively high at the national level, the variable “MCV1 coverage” shows an important relationship in all countries when considered independently, and explains, in four of the five countries, the variance in ICERs between subnational regions. Sanitation, on the other hand, despite its greater variance and its importance in setting the incidence of typhoid among wealth quintiles and subnational regions, played a smaller role. Although sanitation was significant as an individual contributor to the ICERs, it often played a statistically nonsignificant role in explaining the variance in the ICERs, when considered jointly with the other drivers. Poverty does not show a consistent direction, underscoring the complicated, nonlinear relationship between poverty distribution, sanitation, and MCV1 coverage.

Table C‑1. Drivers of cost-effectiveness within each of the five countries in the subnational analysis.

The signs in the parenthesis show the direction of correlation between the model inputs and the ICER of the subnational regions in the parentheses, and the asterisk shows if the relationship is statistically significant according to scaled OLS regression. Negative correlations show that ICERs become lower with higher values of the predictor; that is, that the intervention becomes more favorable with higher values. Positive correlations show that the ICER becomes higher with higher values of the predictor; in other words, that the intervention is less favorable.

|  | Burkina Faso | India | Kenya | Malawi | Nepal |
| --- | --- | --- | --- | --- | --- |
| Individual impact on ICER | MCV1 (+)*  Sanitation (-)  Poverty (+) | MCV1 (+)*  Poverty (-)  Sanitation (+) | MCV1 (+)*  Sanitation (+)*  Poverty (+) | Poverty (-)*  MCV1 (+)*  Sanitation (+) | MCV1 (+)*  Poverty (+)*  Sanitation (+)* |
| Joint impact on ICER | Sanitation (+)  MCV1 (+)  Poverty (-) | MCV1 (+)*  Poverty (+)  Sanitation (+)* | Poverty (+)*  MCV1 (+)*  Sanitation (+)* | Poverty (-)*  MCV1 (+)*  Sanitation (-) | MCV1 (+)*  Poverty (+)*  Sanitation (+) |

Abbreviations: ICER, incremental cost-effectiveness ratio; MCV, measles-containing vaccine.

# References

1. Pitzer, V.E., et al., Predicting the impact of vaccination on the transmission dynamics of typhoid in South Asia: a mathematical modeling study*.* *PLoS Neglected Tropical Diseases*, 2014. **8**(1): p. e2642. 10.1371/journal.pntd.0002642

2. Pitzer, V.E., et al., Mathematical Modeling to Assess the Drivers of the Recent Emergence of Typhoid Fever in Blantyre, Malawi*.* *Clinical Infectious Diseases*, 2015. **61 Suppl 4**(Suppl 4): p. S251-8. 10.1093/cid/civ710

3. Saad, N.J., et al., The impact of migration and antimicrobial resistance on the transmission dynamics of typhoid fever in Kathmandu, Nepal: A mathematical modelling study*.* *PLoS Neglected Tropical Diseases*, 2017. **11**(5): p. e0005547. 10.1371/journal.pntd.0005547

4. Phillips, M.T., et al., Cost-effectiveness analysis of typhoid conjugate vaccines in an outbreak setting: a modeling study*.* *BMC Infectious Diseases*, 2023. **23**(1): p. 143. 10.1186/s12879-023-08105-2

5. Burrows, H., et al., Comparison of model predictions of typhoid conjugate vaccine public health impact and cost-effectiveness*.* *Vaccine*, 2023. **41**(4): p. 965-975. 10.1016/j.vaccine.2022.12.032

6. Antillón, M., et al., Cost-effectiveness analysis of typhoid conjugate vaccines in five endemic low- and middle-income settings*.* *Vaccine*, 2017. **35**: p. 3506-3514. 10.1016/j.vaccine.2017.05.001

7. Bilcke, J., et al., Cost-effectiveness of routine and campaign use of typhoid Vi-conjugate vaccine in Gavi-eligible countries: a modelling study*.* *The Lancet Infectious Diseases*, 2019. **19**: p. 728-739. 10.1016/S1473-3099(18)30804-1

8. Birger, R., et al., Estimating the effect of vaccination on antimicrobial-resistant typhoid fever in 73 countries supported by Gavi: a mathematical modelling study*.* *The Lancet Infectious Diseases*, 2022. **22**(5): p. 679-691. 10.1016/S1473-3099(21)00627-7

9. Antillón, M., et al., The burden of typhoid fever in low- and middle-income countries: A meta-regression approach*.* *PLoS Neglected Tropical Diseases*, 2017. **11**: p. e0005376. 10.1371/journal.pntd.0005376

10. Global Burden of Disease Study. Global Burden of Disease Study 2010 (GBD 2010) Results by Cause 1990-2010. 2012 [Accessed 5 April 2024]; Available from: <http://ghdx.healthdata.org/record/global-burden-disease-study-2010-gbd-2010-results-cause-1990-2010>

11. Brockett, S., et al., Associations among Water, Sanitation, and Hygiene, and Food Exposures and Typhoid Fever in Case-Control Studies: A Systematic Review and Meta-Analysis*.* *American Journal of Tropical Medicine and Hygiene*, 2020. **103**(3): p. 1020-1031. 10.4269/ajtmh.19-0479

12. Verguet, S., et al., Public finance of rotavirus vaccination in India and Ethiopia: An extended cost-effectiveness analysis*.* *Vaccine*, 2013. **31**: p. 4902-4910. 10.1016/j.vaccine.2013.07.014

13. United Nations, Department of Economic and Social Affairs, and P. Division. World Population Prospects 2022. 2022 [Accessed 5 April 2024]; Available from: <https://population.un.org/wpp/Download/Standard/CSV/>.

14. Hornick, R.B., et al., Typhoid Fever: Pathogenesis and Immunologic Control*.* *The New England Journal of Medicine*, 1970. **283**(13): p. 686-691. 10.1056/nejm197009242831306

15. Ames, W.R. and M. Robins, Age and Sex as Factors in the Development of the Typhoid Carrier State, and a Method for Estimating Carrier Prevalence*.* *American Journal of Public Health and the Nation's Health*, 1943. **33**(3): p. 221-30. 10.2105/ajph.33.3.221

16. Carey, M.E., et al., Global diversity and antimicrobial resistance of typhoid fever pathogens: Insights from a meta-analysis of 13,000 Salmonella Typhi genomes*.* *eLife*, 2023. **12**: p. e85867. 10.7554/eLife.85867

17. John, J., et al., Burden of Typhoid and Paratyphoid Fever in India*.* *The New England Journal of Medicine*, 2023. **388**(16): p. 1491-1500. 10.1056/NEJMoa2209449

18. Garrett, D.O., et al., Incidence of typhoid and paratyphoid fever in Bangladesh, Nepal, and Pakistan: results of the Surveillance for Enteric Fever in Asia Project*.* *The Lancet Global Health*, 2022. **10**(7): p. e978-e988. 10.1016/S2214-109X(22)00119-X

19. Meiring, J.E., et al., Burden of enteric fever at three urban sites in Africa and Asia: a multicentre population-based study*.* *The Lancet Global Health*, 2021. **9**(12): p. e1688-e1696. 10.1016/s2214-109x(21)00370-3

20. Marchello, C.S., M. Birkhold, and J.A. Crump, Complications and mortality of typhoid fever: A global systematic review and meta-analysis*.* *Journal of Infection*, 2020. **81**(6): p. 902-910. 10.1016/j.jinf.2020.10.030

21. Mejia, N., et al., Typhoid and Paratyphoid Cost of Illness in Nepal: Patient and Health Facility Costs From the Surveillance for Enteric Fever in Asia Project II*.* *Clinical Infectious Diseases*, 2020. **71**(Suppl 3): p. S306-s318. 10.1093/cid/ciaa1335

22. Mejia, N., et al., Typhoid and Paratyphoid Cost of Illness in Bangladesh: Patient and Health Facility Costs From the Surveillance for Enteric Fever in Asia Project II*.* *Clinical Infectious Diseases*, 2020. **71**(Suppl 3): p. S293-s305. 10.1093/cid/ciaa1334

23. Mejia, N., et al., Typhoid and Paratyphoid Cost of Illness in Pakistan: Patient and Health Facility Costs From the Surveillance for Enteric Fever in Asia Project II*.* *Clinical Infectious Diseases*, 2020. **71**(Suppl 3): p. S319-s335. 10.1093/cid/ciaa1336

24. Chauhan, A.S., et al., Cost effectiveness of typhoid vaccination in India*.* *Vaccine*, 2021. **39**(30): p. 4089-4098. 10.1016/j.vaccine.2021.06.003

25. Limani, F., et al., Estimating the economic burden of typhoid in children and adults in Blantyre, Malawi: A costing cohort study*.* *PLOS ONE*, 2022. **17**(11): p. e0277419. 10.1371/journal.pone.0277419

26. Carias, C., et al., Economic evaluation of typhoid vaccination in a prolonged typhoid outbreak setting: the case of Kasese district in Uganda*.* *Vaccine*, 2015. **33**(17): p. 2079-85. 10.1016/j.vaccine.2015.02.027

27. Adamou, H., et al., Le fardeau de la perforation typhique de l’intestin grêle au Niger*.* *Journal Sahélien des Sciences de la Santé*, 2021. **001**: p. 131-139.

28. Salomon, J.A., et al., Common values in assessing health outcomes from disease and injury: disability weights measurement study for the Global Burden of Disease Study 2010*.* *The Lancet*, 2012. **380**(9859): p. 2129-2143. 10.1016/S0140-6736(12)61680-8

29. Sur, D., et al., Treatment cost for typhoid fever at two hospitals in Kolkata, India*.* *Journal of Health, Population and Nutrition*, 2009. **27**(6): p. 725-32. 10.3329/jhpn.v27i6.4323

30. Riewpaiboon, A., et al., Cost of illness due to typhoid Fever in Pemba, Zanzibar, East Africa*.* *Journal of Health, Population and Nutrition*, 2014. **32**(3): p. 377-85.

31. Poulos, C., et al., Cost of illness due to typhoid fever in five Asian countries*.* *Tropical Medicine & International Health*, 2011. **16**(3): p. 314-323. 10.1111/j.1365-3156.2010.02711.x

32. Raju, R., et al., Healthcare Utilization Survey in the Hybrid Model of the Surveillance for Enteric Fever in India (SEFI) Study: Processes, Monitoring, Results, and Challenges*.* *The Journal of Infectious Diseases*, 2021. **224**(Supplement_5): p. S529-S539. 10.1093/infdis/jiab371

33. Shakya, M., et al., Phase 3 Efficacy Analysis of a Typhoid Conjugate Vaccine Trial in Nepal*.* *The New England Journal of Medicine*, 2019. **381**(23): p. 2209-2218. 10.1056/NEJMoa1905047

34. Qadri, F., et al., Protection by vaccination of children against typhoid fever with a Vi-tetanus toxoid conjugate vaccine in urban Bangladesh: a cluster-randomised trial*.* *The Lancet*, 2021. **398**(10301): p. 675-684. 10.1016/s0140-6736(21)01124-7

35. Patel, P.D., et al., Safety and Efficacy of a Typhoid Conjugate Vaccine in Malawian Children*.* *The New England Journal of Medicine*, 2021. **385**(12): p. 1104-1115. 10.1056/NEJMoa2035916

36. Yousafzai, M.T. and A.E. Heywood, Typhoid conjugate vaccine: are we heading towards the elimination of typhoid in endemic countries? *The Lancet Global Health*, 2022. **10**(9): p. e1224-e1225. 10.1016/s2214-109x(22)00328-x

37. Patel, P.D., et al., Efficacy of typhoid conjugate vaccine: final analysis of a 4-year, phase 3, randomised controlled trial in Malawian children*.* *The Lancet*, 2024. **403**(10425): p. 459-468. 10.1016/s0140-6736(23)02031-7

38. Nampota-Nkomba, N., et al., Using Typhoid Conjugate Vaccines to Prevent Disease, Promote Health Equity, and Counter Drug-Resistant Typhoid Fever*.* *Open Forum Infectious Diseases*, 2023. **10**(Supplement_1): p. S6-S12. 10.1093/ofid/ofad022

39. Sikorski, M.J. Amidst a global pandemic, Samoa rolls out TCV and other new vaccines. 30 August 2021 [Accessed 5 April 2024]; Available from: <https://www.coalitionagainsttyphoid.org/amidst-a-global-pandemic-samoa-rolls-out-tcv-and-other-new-vaccines/>.

40. Joh, J. and R. Zellweger. TCV mass vaccination campaign in Vanua Levu, Fiji. 3 July 2023 [Accessed 5 April 2024]; Available from: <https://www.coalitionagainsttyphoid.org/tcv-mass-vaccination-campaign-in-vanua-levu-fiji/>.

41. NationMaster. Military Personnel Per Capita. [Accessed 5 April 2024]; Available from: <https://www.nationmaster.com/country-info/stats/Military/Personnel/Per-capita>.
